# Negative selection in tumor genome evolution acts on essential cellular functions and the immunopeptidome

**DOI:** 10.1186/s13059-018-1434-0

**Published:** 2018-05-31

**Authors:** Luis Zapata, Oriol Pich, Luis Serrano, Fyodor A. Kondrashov, Stephan Ossowski, Martin H. Schaefer

**Affiliations:** 1 Genomic and Epigenomic Variation in Disease Group, Centre for Genomic Regulation (CRG), The Barcelona Institute of Science and Technology, Dr. Aiguader 88, 08003 Barcelona, Spain; 2 Centre for Evolution and Cancer, The Institute of Cancer Research, London, UK; 3 Evolutionary Genomics Group, Centre for Genomic Regulation (CRG), The Barcelona Institute of Science and Technology, Dr. Aiguader 88, 08003 Barcelona, Spain; 4 Institute for Research in Biomedicine (IRB Barcelona), The Barcelona Institute of Science and Technology, Baldiri Reixac, 10, 08028 Barcelona, Spain; 5 Design of Biological Systems Group, Centre for Genomic Regulation (CRG), The Barcelona Institute of Science and Technology, Dr. Aiguader 88, 08003 Barcelona, Spain; 6 Universitat Pompeu Fabra (UPF), Barcelona, Spain; 7 Institució Catalana de Recerca i Estudis Avançats (ICREA), Pg. Lluis Companys 23, 08010 Barcelona, Spain; 8 IST Austria (Institute of Science and Technology Austria), Am Campus 1, 3400 Klosterneuburg, Austria; 9 Institute of Medical Genetics and Applied Genomics, University of Tübingen, Tübingen, Germany

**Keywords:** Tumor evolution, Negative selection, Cancer-essential genes, Neoepitopes, Cancer immunology

## Abstract

**Background:**

Natural selection shapes cancer genomes. Previous studies used signatures of positive selection to identify genes driving malignant transformation. However, the contribution of negative selection against somatic mutations that affect essential tumor functions or specific domains remains a controversial topic.

**Results:**

Here, we analyze 7546 individual exomes from 26 tumor types from TCGA data to explore the portion of the cancer exome under negative selection. Although we find most of the genes neutrally evolving in a pan-cancer framework, we identify essential cancer genes and immune-exposed protein regions under significant negative selection. Moreover, our simulations suggest that the amount of negative selection is underestimated. We therefore choose an empirical approach to identify genes, functions, and protein regions under negative selection. We find that expression and mutation status of negatively selected genes is indicative of patient survival. Processes that are most strongly conserved are those that play fundamental cellular roles such as protein synthesis, glucose metabolism, and molecular transport. Intriguingly, we observe strong signals of selection in the immunopeptidome and proteins controlling peptide exposition, highlighting the importance of immune surveillance evasion. Additionally, tumor type-specific immune activity correlates with the strength of negative selection on human epitopes.

**Conclusions:**

In summary, our results show that negative selection is a hallmark of cell essentiality and immune response in cancer. The functional domains identified could be exploited therapeutically, ultimately allowing for the development of novel cancer treatments.

**Electronic supplementary material:**

The online version of this article (10.1186/s13059-018-1434-0) contains supplementary material, which is available to authorized users.

## Background

The evolution of human cancers is similar in nature to the evolution of non-recombining unicellular microorganisms [1, 2]. The hallmarks of evolution include positive selection towards increasing the frequency of tumor-beneficial mutations and negative selection towards preventing the accumulation of harmful ones. Since the early 1970s, studies have explored an evolutionary model of tumor development focusing on the role of positive selection to identify genes that are relevant for malignant transformation and tumor progression [3, 4]. Somatic mutations conferring a selective advantage affect specific cellular pathways and processes involving cancer hallmarks such as increased proliferative capacity, suppression of cell cycle control, and escape from immune surveillance [5].

Recent cancer studies have identified cancer-causing or driver genes by detecting signals of positive selection [6, 7]. Through large-scale sequencing of cancer patients, the International Cancer Genome Consortium (ICGC) and The Cancer Genome Atlas (TCGA) initiatives have made thousands of cancer exomes available [8–11]. Consequently, methods exploiting data from these initiatives have revealed an extensive landscape of somatic point mutations in driver genes across tumor types (mutational drivers). The number of cancer genes undergoing positive selection of somatic point mutations identified by in silico approaches is in the range of approximately 100–500 [6, 12–14].

The existence of negative selection acting on cancer genomes is a highly controversial topic: several studies have questioned the presence of strong negative selection [15–17]. In particular, a recent study concluded that negative selection would be almost undetectable outside homozygous loss of essential genes [18]. This is surprising given that cancer essentiality screens have identified fitness altering genes even in diploid regions or incomplete knockdown conditions [19]. Accordingly, negative selection has been identified in particular regions or domains: e.g. in transcription factor binding motifs [20]; membrane proteins [21]; against nonsense mediated decay-inducing mutations in onco- and essential genes [22]; in splicing-associated sequences [23]; and within hemizygous regions [24]. Such recent experimental and computational identification of cancer vulnerabilities raises the question of why exome-wide approaches employing tools from the field of population genetics [17, 18] have only identified a small number of negatively selected genes?

On one hand, the recessive nature of novel deleterious mutations prevent negative selection from acting in most genes [18, 25], low synonymous mutation rates make positive selection more easily detectable when testing against neutrality than negative selection [26], and mutational data availability forces the use of specialized approaches dealing with noise in hypomutated regions [17]. On the other hand, germline variants erroneously labelled as somatic mutations [18] and mutational processes could introduce technical bias [27], thereby falsely suggesting negative selection. Here, we have developed a method that uses *d*_N_/*d*_S_, the ratio of non-synonymous substitutions to synonymous substitutions per site, to detect genes under selection [28]. We present a comprehensive study that addresses the extent and global properties of negative selection across tumor types using strictly filtered whole exome sequencing data. Notably, functional properties of negative selection in tumor evolution become evident when a relaxed empirical cut-off for selection is used. Finally, we demonstrate that immune-mediated negative selection (1) acts on the MHC-exposed regions of native epitopes and (2) correlates with the cytolytic activity across tumor types.

## Results

### A somatic substitution bias-corrected *d*_N_/*d*_S_ measure reveals negative selection in cancer exomes

**Fig. 1:**
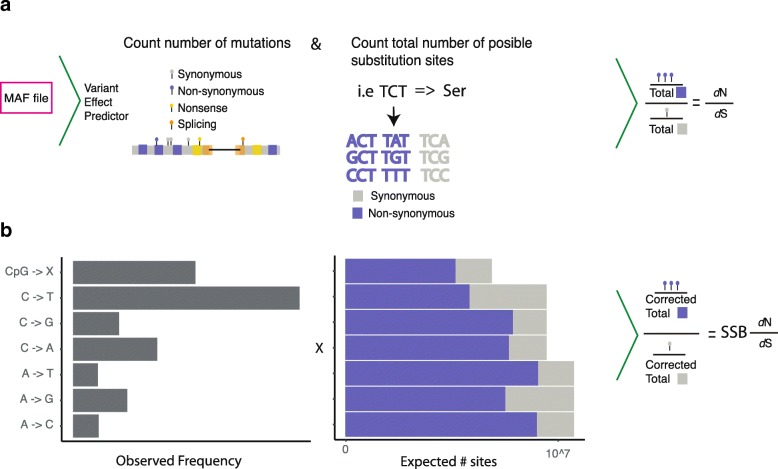
Discovery of negatively selected genes. *Schematic* workflow for using ICGC/TCGA data to detect negatively selected genes. **a** Workflow for calculating *d*_*N*_*/d*_*S*_ using counts of somatic mutations and the human coding sequence without using a substitution model. **b** Descriptive values using mutational data to correct for mutation frequencies. The substitution model exemplified uses seven substitution types, but any other model could be implemented. The observed frequency of substitutions is used to correct for the expected number of sites in all transcripts to calculate a corrected value of *d*_*N*_*/d*_*S*_ (SSB- *d*_*N*_*/d*_*S*_)

To identify genes under negative selection in cancer we analyzed 7546 individual samples across 26 tumor types (Pancan26, Additional file 1: Table S1) using a somatic substitution bias (SSB)-corrected *d*_N_/*d*_S_ measure (SSB-*d*_N_/*d*_S_). Our method corrects *d*_N_/*d*_S_ using a model of seven somatic-specific substitution frequencies (Fig. 1, see “Methods”). Based on the SSB-*d*_N_/*d*_S_ values, we applied a stringent statistical test revealing 39 genes under exome-wide significant selection [29] (Q < 0.1, Table 1, Additional file 2: Table S2). Among the 39 significantly selected genes, we found 14 to be under positive selection and 25 to be under negative selection (Table 1, Additional file 1: Table S3). All 14 of the significant positively selected genes were previously found as being frequently mutated in cancer. Given the 100s of known cancer genes under positive selection, we wondered if the high precision comes at the price of low recall. Indeed, upon comparing the precision and recall to a previously published gold standard of cancer drivers [30], we found that only a substantial relaxation of the false discovery rate (FDR) cut-off leads to a recovery of most known cancer drivers (Additional file 3: Figure S1). Therefore, to test if the number of negatively selected genes was also underestimated we simulated sets of negatively selected genes (Additional file 4). In a dataset of one million somatic mutations, similar to the pan-cancer dataset used, our simulation estimated a recall of ~ 34% for negatively selected genes (Additional file 3: Figure S2). Thus, we expect the total number of negatively selected genes in the pan-cancer analysis to be ~ 75. We also noted that at least 3 million somatic mutations are necessary to reach a recall of 75%.

**Table 1:** Genes under significant selection

Gene name	*d*_N_/*d*_S_	*Q* value
*AP2S1*	0.043	0.0107
*BCL2L12* ^a^	0.093	0.0001
*RALBP1*	0.121	0.0738
*CLDN9*	0.126	0.0625
*GTSF1L* ^b^	0.178	0.0501
*ZDHHC3*	0.255	0.0625
*DECR1*	0.286	0.0341
*HLA-DOA*	0.291	0.0581
*TMEM214*	0.328	0.0408
*GRID2IP* ^a^	0.331	0.0107
*DAGLB*	0.338	0.0241
*GFRA3*	0.351	0.0532
*TERT*	0.368	0.0007
*KRI1*	0.372	0.0408
*ZBTB7C*	0.379	0.0073
*NPSR1*	0.404	0.0241
*AP1B1*	0.41	0.0387
*WISP1*	0.421	0.0408
*MCM2*	0.434	0.0581
*XKR6*	0.471	0.0802
*CYFIP1*	0.475	0.0317
*TYK2*	0.521	0.0802
*EPPK1*	0.591	0.0073
*CACNA1S*	0.625	0.0632
*TECTA* ^b^	0.626	0.0209
*FGFR2*	2.36	0.0802
*ERBB3*	2.523	0.0428
*KEAP1*	2.701	0.048
*CTNNB1*	3.344	0
*SMAD4*	3.976	0.0387
*PTEN*	4.756	0
*FBXW7*	5.577	0
*HRAS*	5.636	0.0802
*PIK3CA*	5.928	0
*SPOP*	6.89	0.0016
*BRAF*	9.782	0
*TP53*	10.304	0
*IDH1*	21.589	0
*KRAS*	25.681	0

Genes with *d*_N_/*d*_S_ < 1 are under negative selection

^a^Genes with signals of negative selection potentially influenced by germline variants or positive selection on silent mutations: *GRID2IP* has 17 synonymous somatic mutations having an EXAC allele frequency > 0.001, *BCL2L12* has a silent mutation cluster.

^b^Not significant after removing non-diploid regions

To assess our SSB-correction strategy, we confirmed that the aggregated value of SSB-*d*_N_/*d*_S_ across all genes (global *d*_N_/*d*_S_) was close to one for every type of tumor (Additional file 1: Table S4), with only mildly inflated *P* values (Additional file 3: Figure S3), and in agreement with previous observations [31]. In comparison, results obtained without correcting for mutation signature showed strongly inflated *P* values. SSB correction improved results for most types of tumors, notably for skin melanoma in which the C- > T signature is the most dominant substitution (Additional file 3: Figure S3). We next asked if expanding our initial SSB-correction strategy using seven substitution types (SSB7) to a model accounting for the full trinucleotide sequence context of the mutation and the strand (SSB192) would further improve the results (see “Methods”). To this end, we repeated the simulation of genes under selection to estimate precision and recall for SSB7 and SSB192 (Additional file 3: Figures S2 and S4, Additional file 4). We found no significant performance gain for the identification of negatively selected genes and only a slightly improved precision for the identification of positively selected genes using SSB192 (Additional file 3: Figure S2). In addition, distributions of *d*_N_/*d*_S_ values per gene for SSB7 and SSB192 methods were highly correlated (*r* = 0.98) and both have a mean and median close to one (Additional file 4: Figure S4). Thus, in our study we refer to SSB7-*d*_N_/*d*_S_ values simply as SSB-*d*_N_/*d*_S_.

A recent study claimed that negative selection in tumor evolution is almost absent [18]. Since both methods used in that study, dNdScv and dNdSloc, and our SSB-*d*_N_/*d*_S_ use *d*_N_/*d*_S_ to detect selection, we compared them using the same pan-cancer dataset. We found that per-gene dNdScv-*d*_N_/*d*_S_ and dNdSloc-*d*_N_/*d*_S_ estimates were well correlated to SSB-*d*_N_/*d*_S_ values (*r* = 0.62 / 0.7, Additional file 3: Figure S5A and B) and this correlation was even higher in the set of significant genes (dNdScv to SSB-*d*_N_/*d*_S_, *r* = 0.97). Nonetheless, we observed that the median of dNdScv-*d*_N_/*d*_S_ values per gene differ from the dNdScv-*d*_N_/*d*_S_ value across all genes (global *d*_N_/*d*_S_, Additional file 3: Figure S5C), whereas the median of SSB-*d*_N_/*d*_S_ values was similar to the global estimate. In addition, the median of dNdScv-*d*_N_/*d*_S_ values was higher than the median of SSB-*d*_N_/*d*_S_ values and had more genes under significant positive selection in the former. Such discrepancy was amplified when looking at individual tumor types separately (Additional file 3: Figure S6), hinting at a relationship between the number of mutations considered and the power to detect genes under selection. To further investigate the impact of the number of somatic mutations on the performance of each method, we simulated a neutral dataset. We ran the methods on four datasets having 100 K, 300 K, 500 K, and 1 M mutations (Additional file 3: Figure S5D). As expected under neutrality, the global *d*_N_/*d*_S_ value for all methods was approximately one. The median dNdScv-*d*_N_/*d*_S_ was higher than one confirming an overestimation of the *per gene* dNdScv-*d*_N_/*d*_S_ values. In comparison, SSB-*d*_N_/*d*_S_ values were tightly distributed around the exome-wide estimate with improving concordance for larger number of analyzed variants, hence increasing the power for detecting negative selection. Additionally, we compared SSB-*d*_N_/*d*_S_ results to a recently published Bayesian approach (CBaSe) for detection of genes under selection [17]. We observed that there is a good agreement between genes detected as being under positive or negative selection by CBaSe and our method (Additional file 3: Figure S7). When running CBaSe on our pan-cancer dataset, five out of nine genes detected as significant by CBaSe were also detected as significant by SSB-*d*_N_/*d*_S_ (*BCL2L12*, *TERT*, *AP2S1*, *KRI1*, *TMEM214*). Nevertheless, the other four significant genes found by CBaSe, and not found by SSB-*d*_N_/*d*_S_, had SSB-*d*_N_/*d*_S_ values smaller than one.

### Negative selection in the context of functional impact and redundancy

**Fig. 2:**
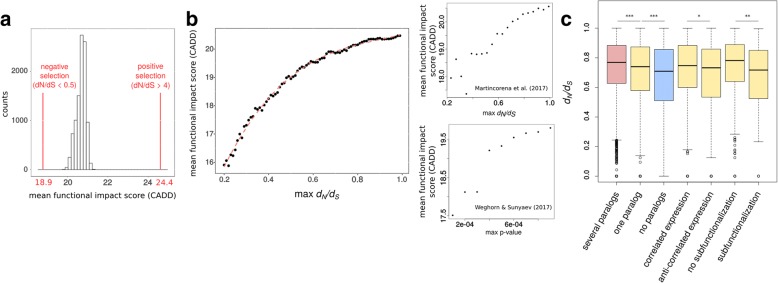
Properties of negatively selected genes in cancer genomes. **a** Missense mutations in negatively selected genes cause less functional impact than missense mutations in non-selected or positively selected genes. The mean functional impact (CADD) score distribution for 10,000 random gene sets of non-selected genes is shown as a reference. The *left red line* indicates the mean functional impact score for a *d*_N_/*d*_S_ threshold of 0.5 (negative selection) and the *right red line* the mean functional impact score for the positively selected genes. **b** Mean functional impact scores are shown for sets of negatively selected genes under different *d*_N_/*d*_S_ thresholds and different methods to calculate negatively selected genes. Furthermore, on single gene level *d*_N_/*d*_S_ ratios and mean functional impact scores are positively correlated (*P* < 10^− 4^; Pearson *r* = 0.61) when considering genes under significant selection. **c** Genes with several paralogs tend to have a higher *d*_N_/*d*_S_ ratio compared to genes with one paralog, which in turn have higher *d*_N_/*d*_S_ values than genes with no paralogs. Genes with one paralog show lower *d*_N_/*d*_S_ values if the paralog has an anti-correlated expression (* *P* < 0.05; *** *P* < 0.001)

Genes under positive selection in cancer, also called cancer driver genes, show a bias towards the accumulation of high functional impact mutations [6, 7, 32]. We hypothesized that genes under negative selection show a bias towards the depletion of high functional impact mutations, and that those genes not influenced by selection do not show any bias. In other words, we expect that mutations strongly altering protein function in a gene under negative selection would be removed from the host genetic pool because they will hinder tumor proliferation and thus these genes will only tolerate low or no functional impact mutations. To test this hypothesis, we obtained Combined Annotation-Dependent Depletion (CADD) functional impact scores of somatic mutations [33] in genes without any evidence of selection, genes under strong negative selection, and genes under strong positive selection. We observed that genes displaying a low SSB-*d*_N_/*d*_S_ ratio (from now on *d*_N_/*d*_S_, unless otherwise specified) were depleted in high functional impact mutations compared to those of neutral and positively selected genes (see Fig. 2a.). Moreover, we found that a higher *d*_N_/*d*_S_ threshold increased the mean functional impact score irrespective of the method used to calculate *d*_N_/*d*_S_ (Fig. 2b). In line with our conclusion that stringent statistical filtering likely underestimates the amount of negatively selected genes, we found that only for *d*_N_/*d*_S_ cut-offs above ~ 0.5 the mean functional impact score converges towards those of non-selected genes (Fig. 2b). Therefore, we focused in the analyses of functional and phenotypic properties of negatively selected genes on genes with *d*_N_/*d*_S_ < 0.5 (668 genes if only genes with > 10 mutations are considered) or on how gene properties behave as a function of *d*_N_/*d*_S_.

Compared to single-copy genes, a lower number of the genes that have paralogs are essential in yeast [34] and humans [35]. It is assumed that paralogs provide redundancy and compensate for gene loss, thus leading to relaxed negative selection in organismal evolution [36, 37]. We therefore investigated if there are differences in negative selection between genes with and without duplicates. Indeed, we observed that genes without paralogs undergo stronger negative selection than genes with paralogs (*P* < 10^–16^; Mann–Whitney *U* test; when considering all genes with *d*_N_/*d*_S_ < 1). Moreover, we observed that genes without paralogs are associated with a smaller *d*_N_/*d*_S_ ratio than genes having one paralog (Fig. 2c; *P* < 10^–5^; Mann–Whitney *U* test) and that genes with several paralogs are associated with even higher *d*_N_/*d*_S_ values (*P* < 10^–4^; Mann–Whitney *U* test). We further hypothesized that paralog pairs having correlated expression across tissues or lower degree of subfunctionalization are more likely to compensate for each other compared to paralog pairs having anti-correlated expression patterns [38]. We found that the *d*_N_/*d*_S_ ratio was significantly higher for negatively selected genes having a co-expressed paralog than for those having a paralog with anti-correlated expression (*P* < 0.05; Mann–Whitney *U* test). Additionally, the *d*_N_/*d*_S_ ratio was lower for genes having one paralog with high subfunctionalization (*P* > 0.01; Mann–Whitney *U* test); which we quantified by the similarity in their domain composition. In summary, we demonstrate that negatively selected genes are protected from mutations having high functional impact and that the strength of the negative selection is dependent on the presence of paralogs.

Additionally, we tested if genes under negative selection are phylogenetically more conserved than neutrally selected genes. Indeed, we observed a slightly elevated conservation of negatively selected genes compared to randomly sampled neutrally selected gene sets (*P* = 0.047; permutation test). Positively selected genes showed a higher evolutionary conservation (Additional file 3: Figure S8).

It has been proposed that the low number of negatively selected genes found in cancer is due to a relaxation on purifying selection because of extra copies of the same gene [25]. To test how ploidy affects our results, we repeated our analysis only considering mutations falling into diploid regions of the genome. We found that the correlation between *d*_N_/*d*_S_ values in diploid-only versus all regions was 0.95 (*P* value < 4.1e-7) and 0.83 (*P* value < 7.7e-7) for positively and negatively selected genes (Additional file 3: Figure S9), respectively. However, two genes, *TECTA* and *GTSF1L*, were no longer significantly under negative selection when looking at diploid-only regions. To further validate our list of negatively selected genes we obtained values of haploinsufficiency. We found six of our negatively selected genes being haploinsufficient, e.g. with a pLI score (probability of being loss of function intolerant) > 0.8, including *TERT*. Moreover, *TERT* has been shown experimentally to be haploinsufficient in mice [39].

*TERT* is the gene showing the second most significant signal of negative selection (Table 1, Q < 0.001) and it has been described as an oncogene in cancer progression [40]. *TERT*, a telomerase reverse transcriptase that maintains telomere ends, is currently the only gene known to be upregulated in several tumor types by a mutation in its promoter [41]. This example demonstrates that functions related to the maintenance of viability during malignant transformation are under negative selection, and that negative selection at the level of protein function can coincide with positive selection of regulatory mutations that increase the protein’s abundance. Our results provide evidence of negative selection acting on the coding sequence of *TERT*, ultimately reaffirming its essential role in cancer.

### The functional role of genes under negative selection and their impact on survival

**Fig. 3:**
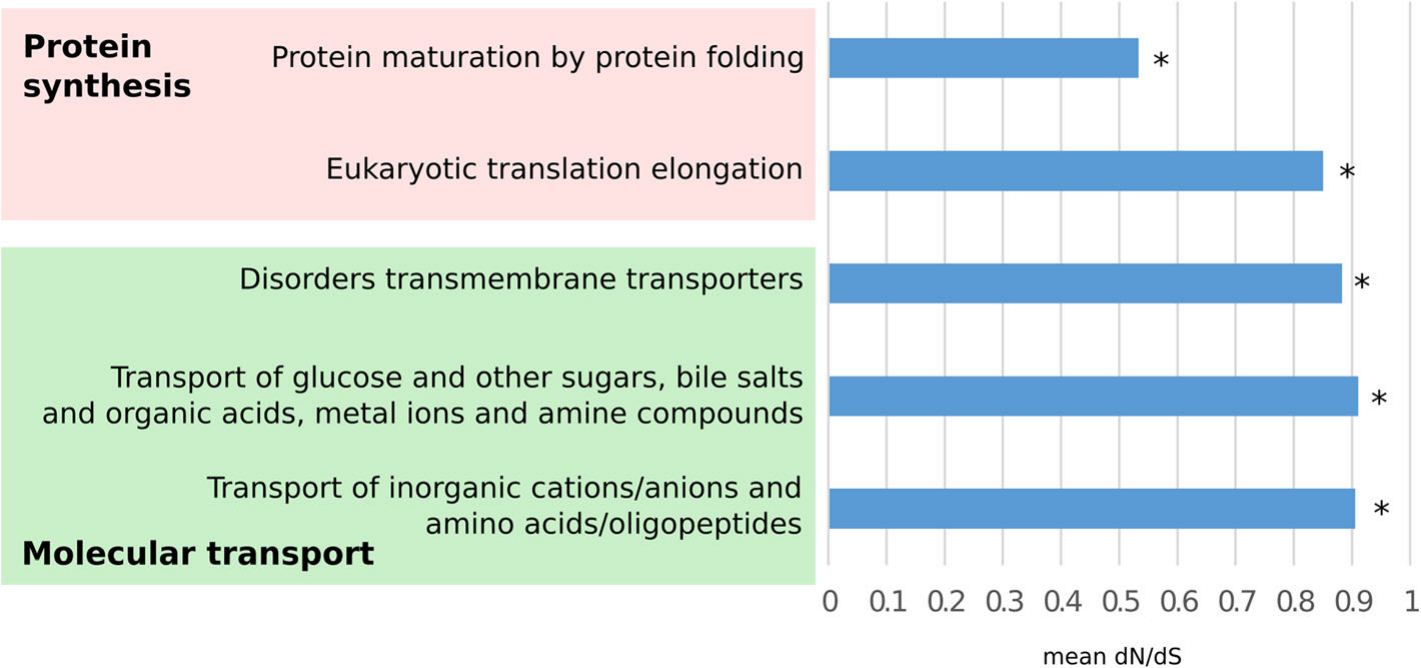
Functional enrichment of negatively selected genes and their impact on survival. Several functions are enriched among negatively selected genes (* Q < 0.1). Most of these functions are related to protein translation and molecular transport

To investigate other cellular processes and functions associated with proteins under negative selection in cancer, we performed a gene set enrichment analysis (GSEA) [42, 43]. Our analysis revealed eight Gene Ontology (GO) terms and five Reactome pathways [44] significantly enriched among negatively selected genes (see Additional file 1: Table S5 for the full list and Fig. 3 for a representative selection). Most enriched terms were related to protein synthesis (e.g. “eukaryotic translation elongation” and “protein maturation by protein folding;” Q < 0.05) or molecule transport (e.g. “transport of glucose and other sugars, bile salts and organic acids, metal ions and amine compounds;” Q < 0.05).

The high number of ribosomal proteins associated with low *d*_N_/*d*_S_ values reflects the general importance of protein synthesis for all living cells and, in particular, for the higher protein synthesis rates of fast growing and dividing cancer cells. In fact, overexpression of translation-promoting proteins has been observed in many cancer types and has been linked to rapid proliferation and malignant transformation [45].

Three Reactome pathways related to molecular transport were enriched among the negatively selected genes (Fig. 3, “Disorders of transmembrane transporters,” “Transport of glucose and other sugars, bile salts and organic acids, metal ions and amine compounds,” “Transport of inorganic cations/anions and amino acids/oligopeptides;” all Q < 0.1). Of these pathways, the 12 members that were under the strongest negative selection (*d*_N_/*d*_S_ < 0.5) were specifically enriched in glucose transport and metabolism (“facilitative Na^+^-independent glucose transporters” and “glucose metabolism;” both Q < 0.05). Specifically, five of the 12 genes, *GCK*, *SLC2A1* (also known as *GLUT1*), *SLC2A8* (also known as *GLUT8*), *CALM3*, and *FGF21*, were involved in at least one of these two pathways. Interestingly, changes in glucose uptake and higher rates of glycolysis (i.e. the Warburg effect) are among the hallmarks of metabolic changes in cancer [46, 47]. Accordingly, several of the aforementioned glucose-related enzymes have been implicated in metabolic reprogramming. For example, the *SLC2A1* glucose transporter is known to be a key mediator of the Warburg effect [48]. Knockdown of *SLC2A1* has been shown to reverse the Warburg effect [49], decrease proliferation, and induce apoptosis in cancer cell lines and mouse xenografts [48, 50, 51]. Moreover, other studies have shown that a high *SLC2A1* expression level is a marker of poor prognosis for several types of cancer [52, 53]. Interestingly, FGF21 stimulates glucose uptake by upregulating SLC2A1 [54].

Additionally, out of all the transport-related genes subjected to strong negative selection, the lactate transporter *SLC16A3* has the lowest *d*_N_/*d*_S_ ratio (*d*_N_/*d*_S_ = 0.34). This gene is essential for metabolic reprogramming in cancer; in clear renal carcinoma cell lines, its silencing has been shown to cause a partial reversion of the Warburg effect through inhibiting the secretion of glycolysis-generated lactate [55]. Accordingly, both the expression of *SLC16A3* and its DNA methylation levels are predictive of patient survival [56].

Next, we investigated which protein complexes were under negative selection (CORUM database [57]). We applied the same GSEA strategy as above and identified three complexes enriched for negatively selected genes (Additional file 1: Table S5). In agreement with the previously described enrichment of translation-related functions, two of the complexes were linked to the ribosome (“Ribosome, cytoplasmic” and “60S ribosomal subunit, cytoplasmic”).

We found the P2X7 signaling complex to be one of the complexes under strongest negative selection (*P* = 0.029; not significant after multiple testing correction). Members of the P2X7 complex are implicated in the control of proliferation and cell survival [58, 59] and previous studies have demonstrated its importance in cancer progression [60–62]. Interestingly, the P2X7 receptor modulates glycolysis by regulating the SLC2A1 glucose transporter [59], which, as discussed above, is also under strong negative selection. We tested if the presence of mutations in either the P2X7 complex or the *SLC2A1* gene was associated with improved prognosis. We considered 15 tumor types for which more than five patients carried a mutation in one of the P2X7 proteins or the *SLC2A1* gene. By definition of negative selection (absence of missense mutations), groups of mutation carriers were typically too small to allow for sufficient statistical power using Kaplan–Meier statistics. Accordingly, no cancer type mutation carriers showed significantly different survival from non-mutation carriers after multiple testing correction. Instead we computed the Cox hazard ratios for each cancer type. In a comparison between mutated and wild-type P2X7 complex groups, we found that the mutated group was associated significantly more often (*P* = 0.035; Binomial test; Additional file 3: Figure S10) with improved prognosis (cox coefficient < − 0.1; 12 cancer types) than with poor prognosis (cox coefficient > 0.1; three cancer types).

Next, we tested whether besides mutation status the expression level of negatively selected genes could be essential for the tumor and thereby influence the survival of affected patients. By considering gene expression we were able to overcome the problem of the small mutation carrier groups. We tested for each negatively selected gene whether low expression was associated with improved patient survival. We identified ten genes with a *d*_N_/*d*_S_ ratio < 0.5 whose expression showed a significant association to survival in at least one tumor type (Q < 0.1; Kaplan–Meier statistics). For nine of these genes (Additional file 3: Figure S11), improved survival was associated with low expression of the gene, a fraction that is higher than expected by chance alone (*P* = 0.025; Binomial test).

### In vitro versus in vivo gene essentiality

We verified whether our negatively selected genes had been identified as essential in recent mutagenesis screens of cancer cell lines [35, 63]. Surprisingly, we did not find a significant overlap—only 16% of the genes with a *d*_N_/*d*_S_ ratio < 0.5 were found to be essential in at least one of the two experimental screens. However, with respect to functional pathways we observed a much better agreement with the mutagenesis screens [35, 63] (Additional file 1: Table S5). For instance, both screens identified RNA processing and translation to be the most strongly enriched function among cancer-essential genes; likewise, we found that fundamental biological processes required for proliferation (e.g. “Translation”) were enriched among the overlapping gene set (i.e. those genes that were identified as cancer-essential and under negative selection; Q < 10^– 10^). In contrast, for the set of genes that are under negative selection but not cancer-essential, the strongest enrichment is for processes that depend on cell-environment interactions (e.g. pathways related to membrane transport and “Cell-Cell communication;” all Q < 0.1). As such, the glucose metabolism-related genes discussed above are only found in this latter set, possibly reflecting the artificial nature of the in vitro environment used for essentiality experiments.

### Immune-mediated negative selection of neoantigens

**Fig. 4:**
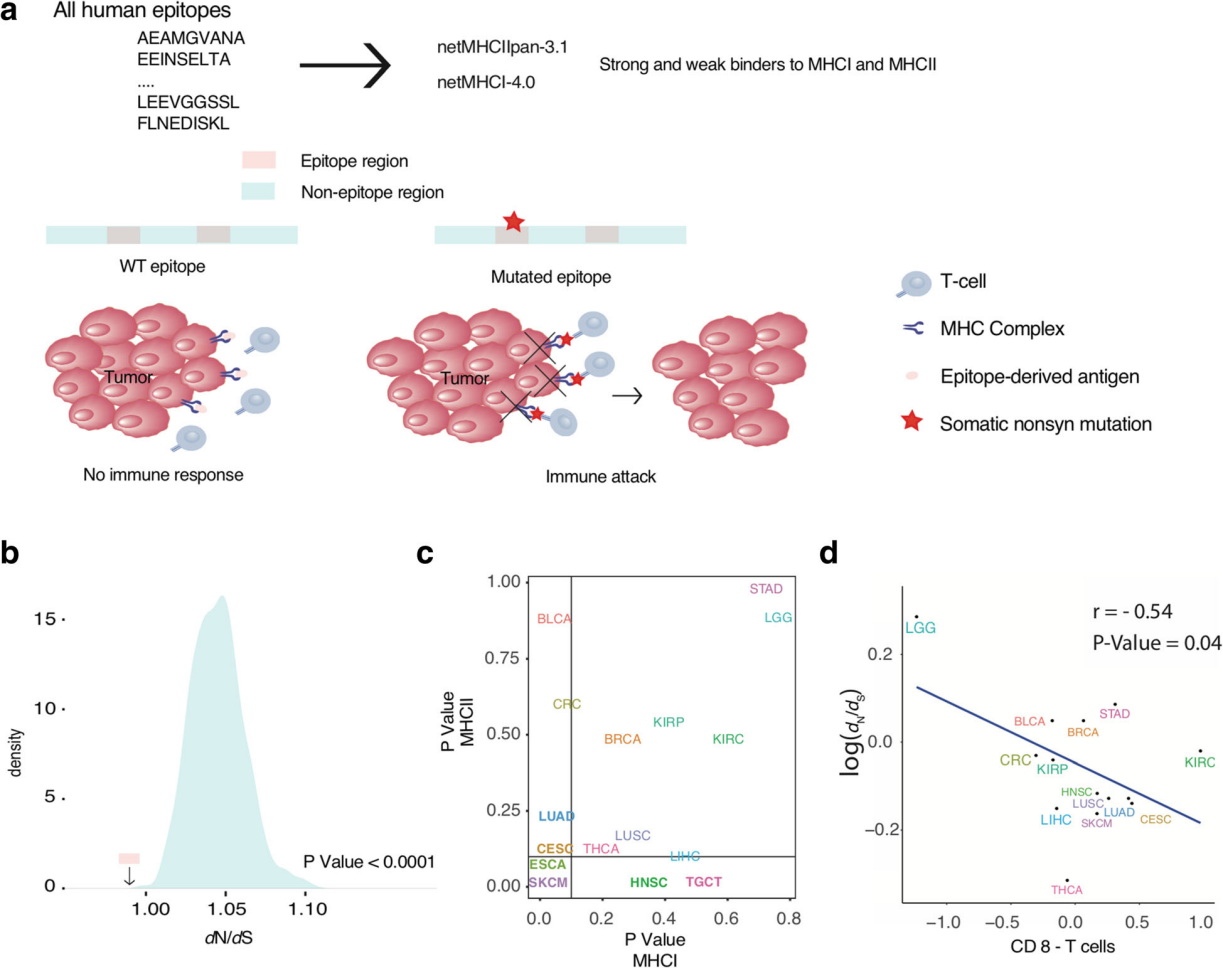
Negative selection of epitopes across multiple tumor types. **a** We assembled lists of epitopes binding to MHC I or MHC II complexes (see “Methods”). Cells carrying mutations on native regions commonly exposed to the immune system are recognized and eliminated by immune cells. We hypothesize that the action of the immune system will leave a signature of negative selection in the cancer genome. Such evidence suggests that tumor cells may escape immune surveillance by acquiring mutations in native non-epitope regions and that native epitope regions become depleted of any high functional impact mutation. **b** The *d*_N_/*d*_S_ ratio for both MHC I- and MHC II-binding epitopes was significantly lower than for a randomized set of non-epitope regions. The *P* value was computed by shuffling the coordinates of equally sized peptides within the same protein. The calculation holds when analyzing specifically patients carrying the HLA-A0201 allele vs patients not carrying this allele. **c** The same calculation was performed separately on MHC I and MHC II epitopes for each tumor type. *Bold* indicates significant when epitope-binding regions from both MHC complexes were combined. See Additional file 1: Table S1 for cancer type abbreviations. **d** *Figure* showing a negative correlation between the *d*_N_/*d*_S_ ratio and the level of immune activity as measured by the quantity of local CD-8 T cells (R is the Pearson correlation coefficient). This suggests that the immune system employs a fundamental tissue-specific mechanism that drives negative selection in tumor evolution

The human immune system is capable of discriminating foreign cells [64] by recognizing the immunopeptidome. This immune response in cancer is (at least partly) mediated by neoantigens or neoepitopes—mutated epitope sequences that, once exposed on the surface of tumor cells by the major histocompatibility complex (MHC), trigger a T-cell immune response (Fig. 4a). We hypothesized that known native epitope sequences would be protected from nonsynonymous mutations. To test this hypothesis, we assembled a consensus list of 13,422 human epitopes by intersecting a large, diverse experimental resource (IEDB) with computational MHC-binding predictions (NetMHC; see “Methods”). We tested if these epitopes were under stronger negative selection compared to the non-exposed regions of the same proteins. Indeed, a significantly lower *d*_N_/*d*_S_ value is associated with the epitope regions across 26 tumor types irrespective of the HLA type of the patient (*P* < 0.0001; permutation test; see “Methods” section; Fig. 4b). As patients differ in their HLA type, we tested the intuition that epitopes bound to more frequent HLA alleles would show stronger negative selection in the cohort as compared to epitopes binding to HLA alleles rarely found in the population. Indeed, the *d*_N_/*d*_S_ of the frequent HLA-A0201-bound immunopeptidome (~ 30% of Caucasian population) was lower than for any of the ten rarest HLA alleles (< 1% of Caucasian population). HLA-B5802 was the only of the rare HLA alleles, for which the binders showed a signal of significant negative selection (see “Methods” for the full list of tested HLA alleles). Next, to strengthen our conclusions we tested the presence of immune mediated negative selection in 2201 patients carrying the allele HLA-A0201 and compared to patients that do not carry this allele (non-HLA-A0201). The SSB-d_N_/d_S_ value for the binding epitopes of HLA-A0201 patients was 0.87 (95% confidence interval [CI] = 0.78–0.97) compared to 0.94 (95% CI = 0.86–1.03) for the binding epitopes of non-HLA-A0201 patients. Additionally, to confirm our observations with an independent and tumor type-specific experimental dataset, we retrieved the HLA-bound peptidome of melanoma cells [65] and repeated the permutation test using melanoma-specific SSB-*d*_N_/*d*_S_ computations. Similarly, we found that these epitopes were also under significant negative selection when compared to random expectation (*P* = 0.005).

We next examined the strength of selection behind MHC I- and MHC II-specific epitopes in individual tumor types (Additional file 1: Table S6). In skin melanoma and esophageal carcinoma, both MHC I- and II-exposed epitopes showed significant negative selection (Fig. 4c). However, while cervical, lung, and bladder cancer only showed a significant negative selection of MHC I-specific epitopes (*P* = 0.024, 0.028, and 0.024, respectively), testicular, and head and neck tumors only showed a significant negative selection of MHC II-specific epitopes (*P* = 0.012 and 0.01, respectively).

As mutation burden (including antigenic mutations) is linked with cytolytic activity of tissue-specific immune infiltrates [66] and different tumor types have a different average mutation burden, we investigated the relationship between tumor type-specific immune activity and the degree of negative selection against neoantigen presentation (Fig. 4d). Five out of 14 features measuring cytolytic activity showed a significant negative correlation with *d*_N_/*d*_S_ ratios over tissues (Fig. 4d, Additional file 1: Table S6B and C, Additional file 3: Figure S12). These results provide evidence that the immune system acts as an important force behind negative selection in tumor evolution, and reveals differences in the way tumors escape the immune response. We found lower grade glioma—a brain tumor that grows in an immune privileged microenvironment where the exposition of antigens does not trigger an immune response—to be among the tumors with the lowest degree of negative selection against neoantigen mutations. On the other hand, cervical tumors showed a strong negative selection of epitopes, which might reflect increased immune response due to papilloma infection preceding carcinogenesis [67].

Interestingly, we identified *HLA-DOA*, a member of the epitope presentation machinery, as one of the most strongly negatively selected genes (*d*_N_/*d*_S_ = 0.29, Q = 0.058). HLA-DOA is the α-subunit of the HLA-DO heterodimer that negatively regulates HLA-DM—the protein responsible for loading peptides on the MHC class II complex [68, 69]. It has been shown that HLA-DO expression and activity diminishes the presentation of self-antigens [70]. Thus, maintaining a functional HLA-DOA protein might form part of the immune escape strategy of cancer cells. The strong negative selection of an MHC class II modulator is compelling given recent evidence highlighting the importance of (MHC class II-binding) CD4(+) T cells in recognizing immunogenic mutations in cancer genomes [71]. Another two genes under significant negative selection are involved in antigen presentation by the MHC complex. AP1B1 (*d*_N_/*d*_S_ = 0.41, Q = 0.039) and AP2S1 (*d*_N_/*d*_S_ = 0.04, Q = 0.01) are members of the clathrin-associated adaptor protein complex 1 (AP-1) and 2 (AP-2), respectively, which are involved in antigen loading onto the MHC class II complex [72, 73]. Interestingly, AP-1 is essential for MHC complex I downregulation and immune escape upon HIV infection [74, 75].

In summary, the presented evidence supports a major role of negative selection in cancer evolution, which has been neglected in most studies, and ultimately challenges the current paradigm of an exclusive role of positive selection in cancer.

## Discussion

With the advent of large-scale tumor sequencing studies, cancer research has focused on the identification of somatic alterations driving tumor malignancy. The central questions behind this line of research have aimed at determining which mutations confer a selective advantage to the cell, which mutations recurrently appear in a particular tumor type, and which mutations have a strong effect on cancer phenotype itself. In contrast, only few recent studies [17, 18, 20–24, 31] have explored whether genes are subjected to negative selection during carcinogenesis. Among the possible effectors of negative selection is the immune system [3], which eliminates cancer cells if they carry somatic mutations that create a neo-antigen or a neo-epitope. Accordingly, a recent study has predicted the likelihood of oncogenic mutations based on the patient-specific MHC-I genotype [76]. Here we demonstrated that immune mediated negative selection acts on native epitope regions using a classic measure of comparative genomics, *d*_*N*_*/d*_*S*_. These studies help to shed light upon the mechanisms underlying immune evasion and provide insights for improving cancer immunotherapies in the future.

We present evidence for extensive negative selection over somatic point mutations in cancer exomes. We exploited a large cancer exome dataset based on 26 tumor types and uncovered a set of 25 genes under negative selection (cancer-essential) and a set of 14 genes under positive selection (cancer drivers). Our results suggest that these numbers are only lower boundaries and we would require around 3 million mutations, which is equivalent to three times as many samples as used in this study, to reach a 75% recall. However, selection acts at different levels [6] and thus negative selection can be tumor type-specific or even patient-specific. It will then take a much larger sequencing effort to reveal cancer genes under tissue-specific or patient-specific negative selection (especially for cancer types with low mutation rates). Here, we make the assumption that different tumor types (as well as subgroups of the same type with different mutation rates) are under common constraints.

Our results challenge the current understanding of cancer evolution—that attributes a dominant role to positive selection [77, 78] or neutral drift [79]. Specifically, we have compared our results to a recently published method demonstrating that the number of negatively selected genes identified depends on the method used. Despite the fact that both methods are based on the *d*_N_/*d*_S_ measure, the method used here and the recently published method by Martincorena et al. [18] have arrived to opposite interpretations of the results. A reason for this difference may lie in the underlying approach to estimate *d*_N_/*d*_S_. Approximate versus maximum likelihood approaches can over- or underestimate *d*_N_/*d*_S_ depending on gene length and sequence divergence [28]. Somatic evolution may represent a special case where sequence divergence is low and/or the number of codons under selection is small. Intriguingly, we find that dNdScv overestimate the median *d*_N_/*d*_S_ in our simulated neutral dataset but apparently not in the neutral dataset simulated in their study. Such discrepancy could be due to the implementation of the simulation: we simulate the SNVs based on context frequency and gene composition, whereas Martincorena et al. simulate the counts per gene based on a binomial model. Despite the differences in the interpretation of the results, we believe that all three methods (dNdScv, cBaSE, and SSB-*d*_N_/*d*_S_) provide complementary strategies for identification of genes under selection in cancer.

The global *d*_N_/*d*_S_ value in cancer genomes is higher than that from germline variation in a human population suggesting a relaxation of negative selection in somatic tissues [80] (Additional file 3: Figure S13). Among the factors contributing to weaker negative selection could be copy number gains in cancer genomes creating redundancy and therefore allowing for the accumulation of mutations [18, 36]. However, when we repeated our analysis in diploid regions only, we could largely reproduce our results suggesting that even though the *d*_N_/*d*_S_ is shifted towards one (neutrality) in cancer genomes it does not imply the absence of negative selection. This is supported by the observation of a depletion of high functional impact mutations in a substantial fraction of the genome (most strongly for genes with a *d*_N_/*d*_S_ < 0.5). Thus, we propose that in addition to *d*_N_/*d*_S_, functional impact of point mutations could be used as a complement for the detection of cancer-essential genes, a strategy that has been applied before for the detection of cancer driver genes [6, 7].

Among the genes under negative selection, we observe a strong enrichment of genes related to translation and molecular transport. This result reflects the high demand of cancer cells for nutrient uptake and protein synthesis due to their increased proliferation. Specifically, we find several glucose transporters and regulators of glycolysis to be under negative selection. Previous studies [48, 50, 51, 53] showed that mutations in this class of genes affect cancer cell viability and therefore disease prognosis. We find both expression and mutation status of negatively selected genes to be related with patient survival, suggesting that these genes could be promising therapeutic targets.

A functional enrichment towards protein synthesis agrees with a previous experimental study that detected cancer-essential genes via quantifying proliferation upon gene knockdown in cancer cell lines [35, 63]. When examining the genes under negative selection but not essential in cancer cell lines, we find many genes involved in processes modulating or depending on the interaction between the cancer cell and its natural environment. As cancer cell lines are strongly adapted to their medium, our patient data-based approach could reveal genes and functions which cannot be experimentally determined as cancer-essential in vitro. This is in line with a recent study demonstrating that in vivo conditions are necessary for detecting environment-specific cancer dependencies in RNAi screens [19].

During the last decade immunotherapy has become an important component of cancer treatment. This type of treatment enhances and promotes the patient’s own immune system to specifically eliminate cancer cells. One established mechanism of the immune response is to recognize antigens that are not present in the normal cells and to eliminate such neoantigen-carrying cells [64]. Cancer cells can acquire somatic mutations within the boundaries of epitopes—the peptides presented to the immune system as antigens—and can therefore be eliminated from the system. Accordingly, the success of immunotherapy is correlated with mutation load [81]. We demonstrate that negative selection acts stronger on native epitope regions than on non-epitope regions, implying that clinically detectable tumors must have escaped surveillance by acquiring copy number alterations or point mutations in non-epitope regions. Although our study provides a proof of concept for the action of immune-mediated negative selection, the existence of suppressed generation of neo-epitopes or the selective effect of reduced epitope binding through mutations remains to be tested. An interesting finding is that MHC-I and MHC-II epitopes are being under different selective pressures in different tumor types. For example, there is an apparent relationship between virus-mediated cancers such as liver or head and neck tumors and negative selection acting on MHC-II specific epitopes. Besides avoidance of mutations in epitope regions, tumors could also rely on suppression of the antigen presentation process itself. Indeed, we show that *HLA-DOA*, a gene that negatively regulates this process, is under strong negative selection.

Several of the genes identified here have previously been implicated in patient survival. Hypothesizing that mutations in cancer-essential genes lead to improved survival, it would thus be interesting to determine whether the presence of mutations in purified genes is correlated with an increase or a decrease in survival. However, genes under negative selection have few substitutions, most of which do not have a strong impact on the function of the protein. The lack of substitutions restricts statistical power when it comes to applying a conventional Kaplan–Meier analysis. However, considering the rapid increase in sequenced cancer exomes and genomes, we expect that in the near future enough data for genes under negative selection will be available to perform this type of analysis.

In summary, we have identified a conservative estimate of 23 genes under significant negative selection. Together with previous literature, our analyses suggest that some of these genes could be potential targets for cancer treatment. One of these, *TERT*, is the only gene identified as a cancer driver because of a recurrently non-coding mutation in its promoter region. We hypothesize that the enhanced expression of *TERT* as a positive selected event imposes a restriction on the coding sequence, ultimately being reflected as a negative selection signal. Potentially positive and negative selection could also act on different protein-coding regions of the same gene (for example positive selection for an activating mutation and negative selection on the remaining regions of the protein to preserve its function). In this case, negative selection would be cancelled out by the effect of positive selection on gene-level and would not be detected by our method.

Our simulation indicates that the increasing availability of sequencing data from individual tumor types will help us to reveal tissue-specific or even patient-specific traces of negative selection. This, in turn, will improve our understanding of cancer-essential functions in different tissues and enable us to develop strategies capable of targeting cancer type-specific essential genes or activating the immune system through optimized modification of epitopes.

## Conclusions

In our work, we demonstrate that despite the extensive amount of neutrally evolving genes in a pan-cancer framework (1) essential cellular functions are under negative selection and (2) there is extensive immune mediated negative selection in specific tumor types.

## Methods

### Tumor data

The TCGA tumor dataset for 25 cancer types was downloaded from the following link: https://www.dropbox.com/sh/fsaxnc3p5jko1ma/AAAlfj4P1aJ0rI7sPAshf4bOa/mafs/tcga_pancancer_dcc_mafs_082115.tar.gz [13]. This consisted of publicly available TCGA somatic mutations files retrieved from Broad GDAC Firehose (date stamp 20,150,824) as described in Kandoth et al. [13]. CLL was obtained from ICGC [82]. Details on how the MAF files were assembled are in the readme document within the compressed file available in synapse. The CLL dataset was obtained from the ICGC-CLL consortium. The 26 tumor types including CLL (“Pancan26”) are described in Additional file 1: Table S1. Population variant allele frequency (VAF), functional impact, and repeat information were obtained from the European Variant Server (EVS), the CADD database [33], and the UCSC genome browser tracks, respectively. Somatic mutations were excluded based on the following criteria: (1) VAF < 0.1; (2) number of reads supporting the alternative allele < 5; (3) EVS frequency ≥1 % ; (4) segmental duplication score > 0.5; (5) UCSC genome browser simple repeat region overlapping the mutation; and (6) allele balance bias (ABB) score≤ 0.7 (Manuscript for ABB score in preparation; see “Methods”). Comparing somatic variants with germline variants having AF > 0.001 in the ExAC database revealed an overlap of < 1%. The only candidate negatively selected gene harboring one potential synonymous germline variant is labelled in the list of selected genes (Table 1). In addition, we removed any gene known to be a false positive in exome studies [13], any gene considered not to be expressed (mean and median RPKM < 1 in 11 or more of the 12 tumor types from Synapse:syn2812925 expression data), and any gene having a ratio for the total number of non-synonymous sites (Na) to synonymous sites (Ns) larger than five. Furthermore, we discarded genes which had zero synonymous and zero non-synonymous substitutions. Using OncodriveCLUST [7], we also labelled genes harboring clusters of potentially functional synonymous SNVs (Q < 0.2). Filtered gene files used for the analysis were uploaded to synapse (syn6115413).

### *d*_N_ and *d*_S_ calculations

All somatic point mutations were annotated using Variant Effect Predictor [83], which provides an Ensembl transcript ID and the respective variant type of the mutation. Missense and nonsense mutations were considered non-synonymous substitutions. Mutations having a different variant type were discarded. In addition, each mutation was assigned to one substitution type (A > T, A > C, A > G, C > A, C > T, C > G, or CpG > N). We then counted all possible substitutions for each transcript present in the MAF file. Finally, we obtained the total number of non-synonymous and synonymous sites for each of the seven substitution types using an approximate method [28]. The ratio of non-synonymous substitutions per non-synonymous sites (*d*_N_) was calculated by dividing the observed number of non-synonymous substitutions by the total number of non-synonymous sites per transcript. Similarly, we obtained the ratio of synonymous substitutions per synonymous sites (*d*_S_) and used these values to calculate the uncorrected *d*_N_/*d*_S_ ratio per transcript.

### Somatic substitution bias (SSB) correction

As different substitution types have different probabilities, we developed a method to correct the number of sites based on the observed frequency of each substitution: A > T, A > C, A > G, C > A, C > T, C > G, and CpG > N (termed somatic substitution bias correction, SSB7) (for details, see Additional file 4). In molecular evolution, selecting a substitution model is key to achieve a correct interpretation of the results. Cancer genomes accumulate mutations more often in CpG sites compared to non-CpG sites [29]. Accordingly, we adjust the model considering six substitutions types by counting changes occurring on CpG sites separately. The observed frequency for these seven substitution types was obtained for each gene per cancer cohort. Next, the relative expected frequency based on the total number of synonymous (Ns) and non-silent (Na) sites was obtained for all human genes (including nonsense and non-synonymous sites as non-silent). Third, we calculated the fold change of the observed versus expected frequencies for each substitution type. Then, we used the obtained fold change to adjust Na and Ns per mutation context per gene. The total per-gene Na and Ns was calculated as the sum across all seven categories. To combine different tumor types, we obtained a pan-cancer Na and Ns based on the fraction of somatic mutations in each tumor type compared to the total number of mutations across 26 tumor types. The full mathematical model is described in Additional file 4. The comparison with the correction taking into account 192 parameters to address the substitution bias and the comparison with the effect of applying different filtering criteria to test the robustness of our set of significantly selected genes is shown in Additional file 3: Figure S2 and S4, and in Additional file 1: Table S7, respectively.

### Statistical analysis

To assess the significance of selection acting on genes we adapted a previously published statistical test (for details, see Additional files) [29]. This test is based on the principle that synonymous somatic mutations are passenger mutations. This enables us to estimate the expected number of non-silent mutations and test against the null hypothesis of neutrality. We calculated *P* values considering the SSB-corrected total number of sites for every gene. Multiple test correction was performed using the Benjamini and Hochberg method. Significant genes were selected based on the adjusted *P* value (Q < 0.1). Significantly positive and negative genes were selected based on the *d*_N_/*d*_S_ measure (> 1 positive, < 1 negative).

### Functional impact scores, paralogs, conservation, and mutation rates of genes under selection

We retrieved the PHRED-scaled CADD scores [33] for all the mutations used to compute the *d*_N_/*d*_S_ ratios. Genes associated with a Q-value < 0.1 were considered to be under selection, while genes associated with a Q-value > 0.8 were considered to be neutral. We computed the mean functional impact score (among non-silent mutations in all genes of the respective sets) for different *d*_N_/*d*_S_ cut-offs for negatively selected genes, for 10,000 randomly sampled neutral gene sets, and for the positively selected genes (*d*_N_/*d*_S_ > 1). *P* values were computed as the number of times the randomized mean functional impact score was more extreme than the observed mean functional impact score. We retrieved paralog information for all human genes from Ensembl via BioMart [84]. We did not apply any filters on sequence similarity between paralog pairs. To test the difference in degree of negative selection between genes with and without paralogs, we first removed genes with a *d*_N_/*d*_S_ ≥ 1. However, the observed differences in *d*_N_/*d*_S_ between genes with and without paralogs are independent of the precise *d*_N_/*d*_S_ cutoff used for filtering (Additional file 3: Figure S14).

To assess the correlation between gene expression and negative selection, we computed the Pearson correlation coefficient between each negatively selected gene associated with a single paralog and the paralog over 53 healthy tissues from GTEx (V6p) [85].

To test differences in negative selection for paralog pairs with high versus low degree of subfunctionalization, we annotated all paralog pairs with InterPro domains [86]. We excluded domains spanning > 25% of a protein as many of the larger annotations are in fact protein family classifications. We then implemented a similarity measure of the domain composition between two proteins as the Jaccard index of the domain annotations of paralog A and paralog B. The difference in *d*_N_/*d*_S_ was significant for all domain composition similarity score cutoffs < 0.38.

For testing conservation differences between genes under positive, neutral, and negative selection, we associated each gene with a measure of phylogenetic conservation [87]. We applied the same randomization strategy as described above for detecting differences in the mutation functional impact between the gene groups. We also computed the mutation rate for each gene ((synonymous + non-synonymous mutations) / transcript length). We did not detect a significant difference between the mutation rates of negatively and positively selected genes; however, both were significantly lower than those of neutrally selected genes (Additional file 3: Figure S15).

### Comparison to dNdScv from Martincorena et al. [18]

The dNdScv tool from Martincorena et al. was obtained from github (https://github.com/im3sanger/dndscv). To compare to our method, the original script was run with options: refdb = “hg19,” sm = “192r_3w,” kc = “cgc81,” cv = “hg19,” max_muts_per_gene_per_sample = 3, max_coding_muts_per_sample = 10,000, use_indel_sites = F, min_indels = 5, maxcovs = 20, constrain_wnon_wspl = T, outp = 3. The latter option allows for three different outputs, a list of per gene results using dnds_cv, a list of per gene results using an alternative method dnds_loc, and a global *d*_N_/*d*_S_ file. In the supplementary figure, both methods are shown for clarity. The input files from pan-cancer and the individual tumor types were adapted from the original MAF file available in synapse. The pan-cancer file in dNdScv format was deposited on synapse syn11617417. In addition, we run dNdScv on four simulated neutral sets having 100 K, 300 K, 500 K, and 1 M somatic mutations, also deposited on synapse. For comparison, a global *d*_N_/*d*_S_ value has been obtained by using all mutations together in SSB, i.e. by considering the whole exome as a single gene. This strategy ensures that the global *d*_N_/*d*_S_ estimate is robust due to being calculated using a large number of non-synonymous and synonymous mutations and comparable to the global *d*_N_/*d*_S_ estimate provided by dNdScv. The median *d*_N_/*d*_S_ was calculated using the *d*_N_/*d*_S_ values of 500 randomly selected genes after removing genes with 0 non-silent or 0 synonymous mutations. Additional file 3: Figure S5 and S6 show the mean value for the median *d*_N_/*d*_S_ and the 95% CI after bootstrapping 100 times.

### Comparison to CBaSe from Weghorn et al. [17]

The list of positively and negatively selected genes and their respective *P* values were obtained from [17]. The list provided two different *P* values: one for testing for negative selection and for testing for positive selection. We assembled a list of positively and a list of negatively selected genes by selecting genes having a Q-value < 0.25 in SSB. Then, we observed the distribution of *P* values obtained for these genes in [17]. Additionally, we used the CBaSe web server to identify negatively selected genes specifically in our pan-cancer call set, using default parameters and allowing the method to choose the best model.

### Functional enrichment

We applied a variant of the GSEA algorithm [42] as described in Schaefer and Serrano [43] to identify enriched GO terms, pathways, and complexes among genes undergoing negative selection. For the analysis, we only considered those GO terms and pathways that were associated with at least 12 genes. Similarly, we only considered those complexes composed of five or more members.

To test the robustness of the observed functional enrichment, and to exclude that less accurate estimates of selection from lowly mutated genes impact the performed analysis, we repeated the GSEA on a reduced gene set containing only those genes with at least ten mutations (silent or missense). We were largely able to reproduce the previously observed functional enrichment: in all three of the functional categories discussed in the manuscript (“protein translation,” “membrane localization and ion transport,” and “metabolism”), several of the previously identified GO or Reactome terms were enriched (e.g. “translation elongation,” “Transport of glucose and other sugars, bile salts and organic acids, metal ions and amine compounds,” and “superoxide metabolic process;” all *P* < 0.01).

To identify processes enriched among the overlapping subset (i.e. cancer-essential genes under negative selection), we used the ConsensusPathDB tool [88]. We considered the mutagenesis screens in K562 and in KBM7 [63]. We computed the enrichment of the two subsets (under negative selection-only and overlap with cancer-essential genes) with respect to the full set of negatively selected genes (all genes with *d*_N_/*d*_S_ < 0.5).

### Survival analysis

Survival analysis was performed using the R package “surv.” For assessing whether P2X7 mutation status affects patient survival, a cox regression model was used to determine the hazard ratio of dying for the group of affected patients compared to the unaffected patients. Then, we used a binomial test to determine if mutations in P2X7 are generally associated with a better prognosis. We excluded those tumors that had an absolute cox coefficient < 0.1 and those tumors for which less than five patients were affected.

To test if expression of genes with low *d*_N_/*d*_S_ affects survival, we considered the 625 genes with the lowest *d*_N_/*d*_S_ (*d*_N_/*d*_S_ < 0.5) having at least ten reported mutations and available expression information in TCGA. We normalized gene expression values by the patient-specific mean expression over all genes. For each gene and cancer type (14 cancer types with > 300 patients), we split the patients into those who displayed higher than median gene expression and those who displayed lower than (or equal to) median gene expression. We then determined if there was a difference in survival between the two patient groups.

To test if the fraction of genes for which low expression was associated with improved survival was higher than expected (among negatively selected genes showing a significant effect on survival), we determined this fraction among genes under neutral selection. As a much lower fraction of those showed a significant association between expression and survival, we had to relax the Q-value threshold to 0.4 resulting in 470 genes. In 54% of those, low expression was associated with improved survival. We therefore set the probability of success parameter p to 0.54 when performing the binomial test.

The nine genes for which low expression was associated with improved survival were *GPR87*, *CACNG2*, *VSIG10L*, *LMX1B*, *MORN5*, *UCMA*, *STRAP*, *FAM109A*, and *C14orf182* (now renamed to *LINC01588*). For the latter the evidence for translation is controversial: while UniProt [89] (accession: B7ZM91) and ProteomicsDB [90] indicate that it is translated into a protein, the new version of HGNC [91] lists it as non-coding (as of 28 August 2017).

### Analysis of negative selection on tumor peptide antigen regions

We retrieved epitope positions of human proteins (66,698 regions) from the IEDB database [92]. We then ran netMHCIIpan-3.1 [93] and netMHC-4.0 [94] (default parameters, alleles HLA-A_0201 and HLA-DRB1_0101) on the total list of peptides retrieved as epitopes. Next, as our final set of candidate epitope regions we extracted 13,422 epitopes labelled by netMHCIIpan-3.1 or netMHC-4.0 as strong (rank < 0.5) or weak binders (rank < 2). We deposited both files used in this study in synapse (id syn11935058). Then, we fused all candidate epitope regions into one super-epitope and the remaining non-epitope parts of the same proteins as one super-non-epitope (proteins with no annotated epitope were not included in the super-non-epitope). Then, we calculated the total number of possible non-synonymous and synonymous substitution sites for both the super -epitope and -non-epitope. The number of observed synonymous and non-synonymous mutations across the tumor dataset for both regions was extracted using bedtools [95]. A SSB7-corrected *d*_N_/*d*_S_ value for the true epitope region was obtained as described above for genes. In order to obtain an exact *P* value, we permutated the true epitope region 1000 times by shifting the coordinates of each region such that it overlapped with a non-epitope region of the same protein and recalculated the *d*_N_/*d*_S_ value. For this permutation we also excluded the first two amino acids of each protein. This resulted in a distribution of *d*_N_/*d*_S_ values for the non-epitope regions matched to the epitope regions. An exact *P* value was obtained directly from the results of the permutation by comparing the true observation to the distribution of the randomized model.

In addition to the allele HLAL-A0201 (present in 30% of the Caucasian population), we also tested for negative selection acting on peptides binding to rare HLA alleles present in < 1% of the Caucasian population. To perform this analysis, we intersected the full list of class I HLA alleles provided in Shukla et al. [96] to the available list of alleles in the netMHC software. From this intersected list, we selected alleles present in < 1% of the population. In addition, we only considered alleles where the bound peptides showed an overlap of < 100 peptides with peptides binding to HLA-A0201. This resulted in a list of ten alleles (HLA-B5802, HLA-A0302, HLA-A3002, HLA-A3301, HLA-B4501, HLA-B5301, HLA-B5401, HLA-B5703, HLA-B7301, HLA-B8101) that were rare and showed no overlap with HLA-A0201 epitopes. Finally, to further strengthen our conclusions we then selected only patients carrying the HLA-A0201 allele and rerun the same permutation analysis described above but using only peptides predicted to bind the MHC-I HLA-A0201 allele. We obtained the HLA types for MHCI regions via TCIA.at [97].

We observed that one of the MHC class II complex genes was under strong negative selection (*HLA-DOA*). Eight *HLA-DOA* alleles exist with minor variations at the nucleotide level but no difference at the amino acid level [98]. We therefore aimed to exclude the possibility that the *d*_N_/*d*_S_ computation might have been affected by misaligned reads. As such, we tested if any of the mutations we used for the *d*_N_/*d*_S_ calculation were identical to inter-allele variation. As this was not the case, we were able to conclude that the reported mutations are likely real and not an artefact of misaligned reads.

#### Cytolytic activity

We obtained a detailed list of measures of cytolytic activity for TCGA patients from Rooney et al. [67]. This list includes amounts of B cells, CD4 regulatory T cells, CD8 T cells, macrophages, neutrophils, NK cells, pDCs, MHC Class I, co-stimulation APC, co-stimulation T cells, co-inhibition APC, co-inhibition T cell, type-I IFN response, type-II IFN response, and a global measure of cytolytic activity. We estimated the Pearson correlation between the mean of these measures per tumor type and the *d*_N_/*d*_S_ values obtained during the analysis of the MHCI, the MHCII, and both epitopes combined.

#### Analysis on diploid-only regions

We downloaded the normalized copy number segment means (level 3 TCGA data) calculated from SNP array data for those patients used in this study. Next, we identified the regions of those patients with segment means between − 0.01 and 0.01 (diploid regions); we removed all somatic mutations outside of those diploid regions, resulting in ~ 700 K somatic mutations. We then calculated SSB-*d*_*N*_*/d*_*S*_ as stated previously and plotted the correlation between *d*_N_/*d*_S_ values in diploid-only regions and all regions for negatively and positively selected genes.

## Additional files

Additional file 1: This document contains additional supporting evidence presented as supplemental tables. (XLSX 50 kb)

Additional file 2: This document contains the full list of genes with their respective significance and *d*_N_/*d*_S_ values. (TXT 4499 kb)

Additional file 3: This document contains additional supporting evidence presented as supplemental figures. (DOCX 3288 kb)

Additional file 4: This document contains the mathematical description of the method used for the context correction. It also contains the description of the simulation, the benchmarking, and the ABB score filtration. (DOCX 19 kb)

Additional file 5: Reviewer reports and Author’s response to reviewers. (DOCX 53 kb)
