# Supplementary material for: Negative selection in tumor genome evolution acts on essential cellular functions and the immunopeptidome
Source: Genome Biol. 2018 May 31;19:67. doi: 10.1186/s13059-018-1434-0 (PMC5984361; doi:10.1186/s13059-018-1434-0)
Supplement: Supplementary file 3 — This document contains additional supporting evidence presented as supplemental figures. (DOCX 3288 kb) [file 13059_2018_1434_MOESM3_ESM.docx]

**Supplemental material**

**Negative selection in tumour genome evolution acts on essential cellular functions and the immunopeptidome**

**Luis Zapata, Oriol Pich, Luis Serrano, Fyodor A. Kondrashov, Stephan Ossowski & Martin H. Schaefer**

**Supplemental Figures**


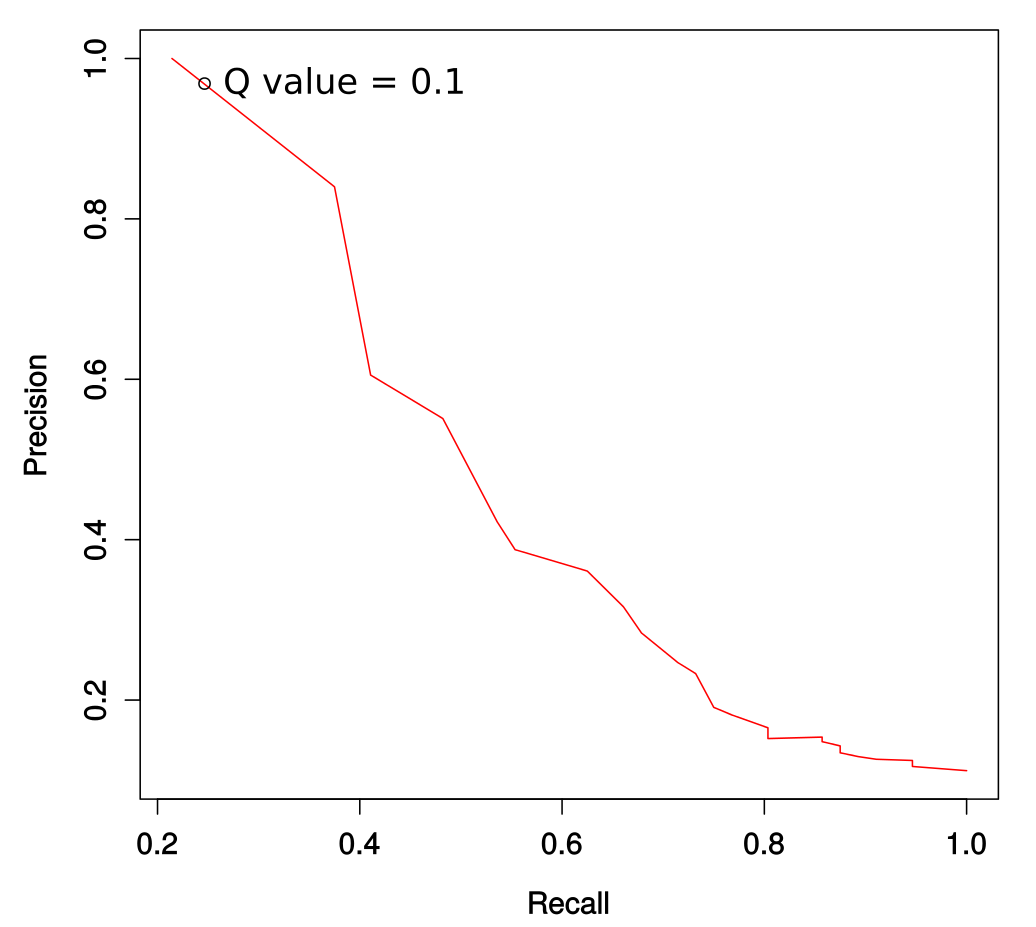


**Figure S1. Performance in reproducing known cancer driver genes.**

We retrieved a list of 260 previously identified genes under positive selection (Lawrence et al. 2012). For the 500 genes with the highest dN/dS ratio, we computed precision and recall for varying significance levels. The performance of the threshold used in this study (Q < 0.1) is indicated.

**Figure S2. Simulation of positively and negatively selected genes.** Precision and recall of SSB7 and SSB192 for positive and negatively selected genes using 100K, 300K, 500K, 1M, and 3M of simulated somatic mutations.


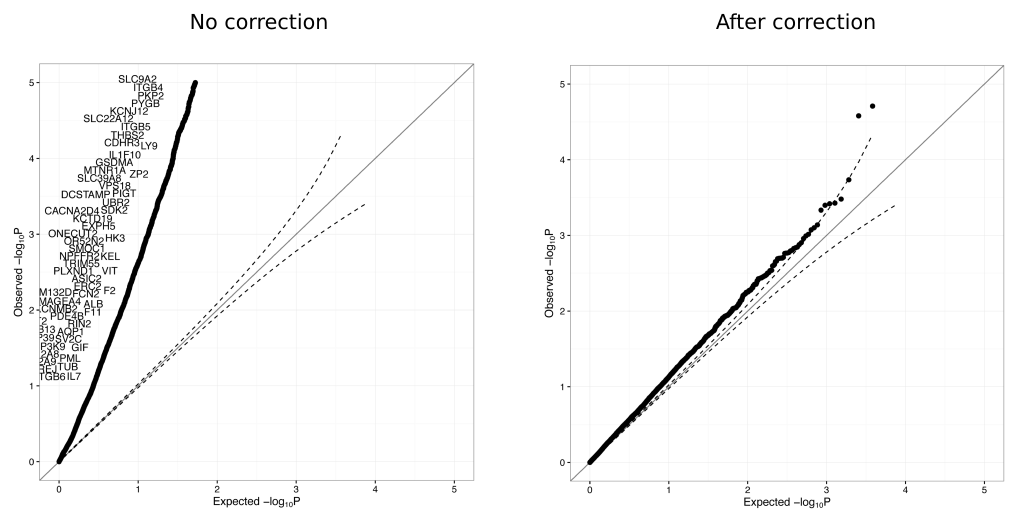


**Figure S3. Effect of Somatic Substitution Bias correction on melanoma using 7 parameters (SBB7).** QQ plot distribution for P-values before (left) and after (right) SBB7 correction on Skin Melanoma samples.

**Figure S4. dN/dS comparison between the uncorrected, SSB7-corrected and SSB192-corrected datasets.** A) dN/dS distribution for uncorrected, SSB7, and SSB192. The dot indicates the mean and the line the median. B) Correlation of dN/dS values for SSB7 versus SSB192 (each dot is one gene).

**Figure S5. dN/dS comparison between the method published in Martincorena et al 2017 and SSB corrected dN/dS.** Correlation plot between obtained dN/dS values in the pancancer dataset analyzed in this work and A) dnds_cv and B) dnds_loc, which are the two methods published in Martincorena et al 2017. C) (Vertical lines) Per gene - Median dNdS distribution (using the mean 95% CI after 100 iterations and sampling 500 genes at each iteration) plots for the three methods in the pancancer dataset and (Horizontal lines) global dN/dS estimates by looking at all mutations together. D) Same as C but using a simulated neutral set having 100K, 300K, 500K, and 1M somatic mutations. The numbers below the vertical lines show the number of significantly positive (+) or negative (+) selected genes for each method.

**Figure S6. Log values of per gene dN/dS.** The mean of the median and the 95% confidence intervals were calculated by bootstrapping 100 times the missense dN/dS of 500 genes each time. In red the median dN/dS obtained running dNdScv and in blue results from the SSB method. For simplicity, lines when the median dN/dS was above 5 were omitted.

**Figure S7. P-Values distribution of Weghorn et al 2017 for genes under negative and positive selection defined by dN/dS.** Upper two plots show the distribution of the ‘N S’ and ‘P S’ P-values for a set of negatively selected genes. The same is shown for a set of positively selected genes in the bottom two plots. The P-values reported in Weghorn et al confirm the dN/dS predictions for a set of genes having q values less than 0.25.


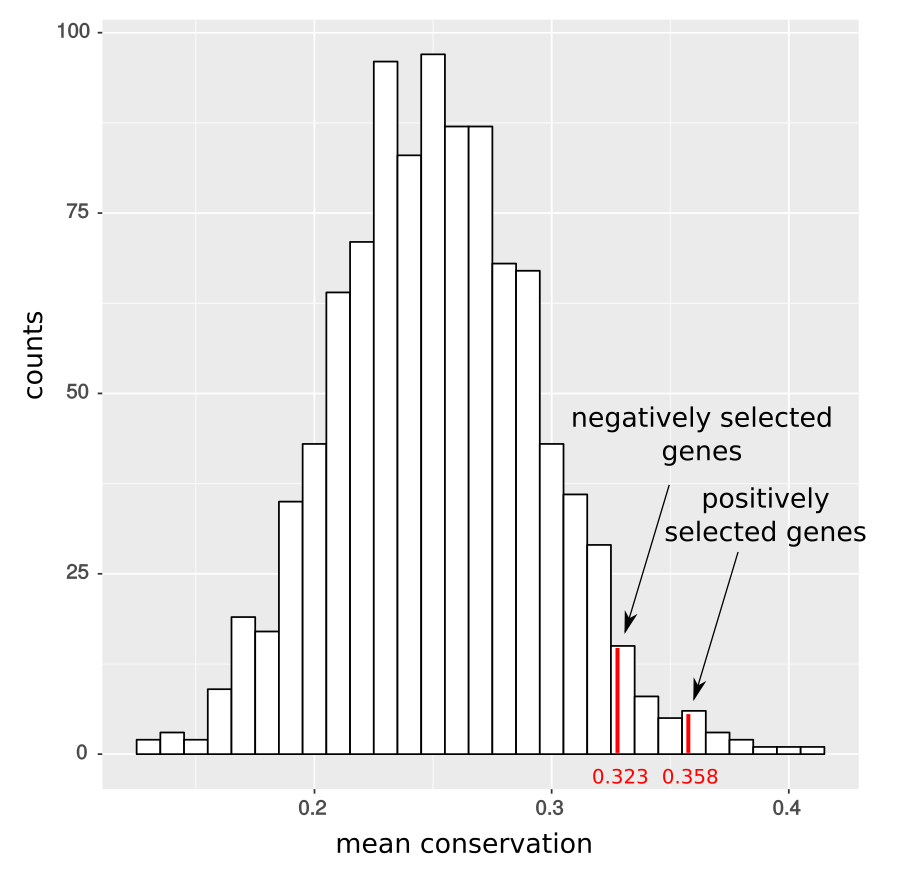


**Figure S8. Phylogenetic conservation of negatively selected genes.**

We randomly sampled sets of neutrally selected genes and computed their mean conservation. Negatively selected genes are more conserved than neutrally selected genes (P = 0.047; randomization test).

**
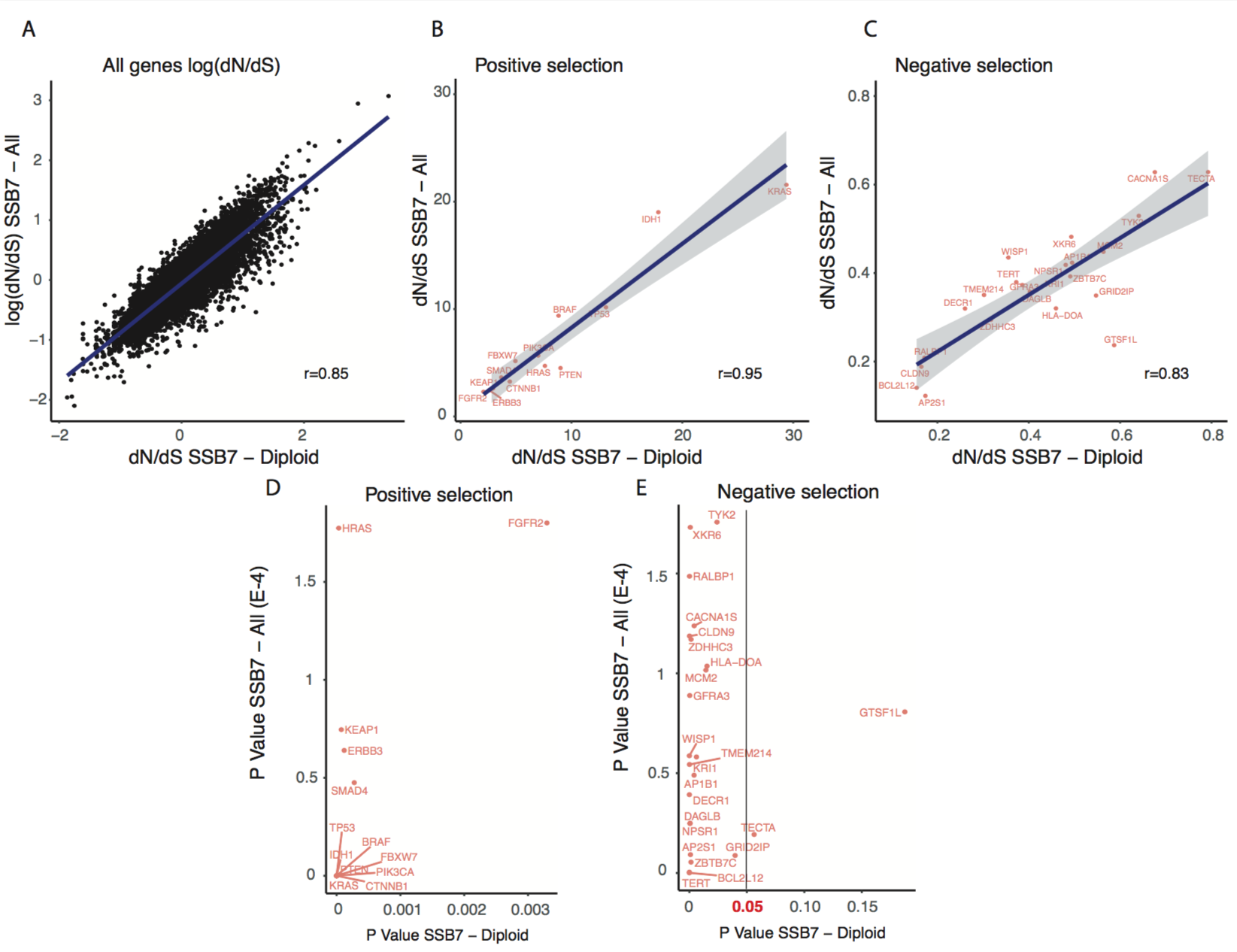
**

**Figure S9. dN/dS values for diploid-only and all regions.** Correlation plots for dN/dS values between diploid-only regions and all regions for A) all, B) positively selected, and C) negatively selected genes.


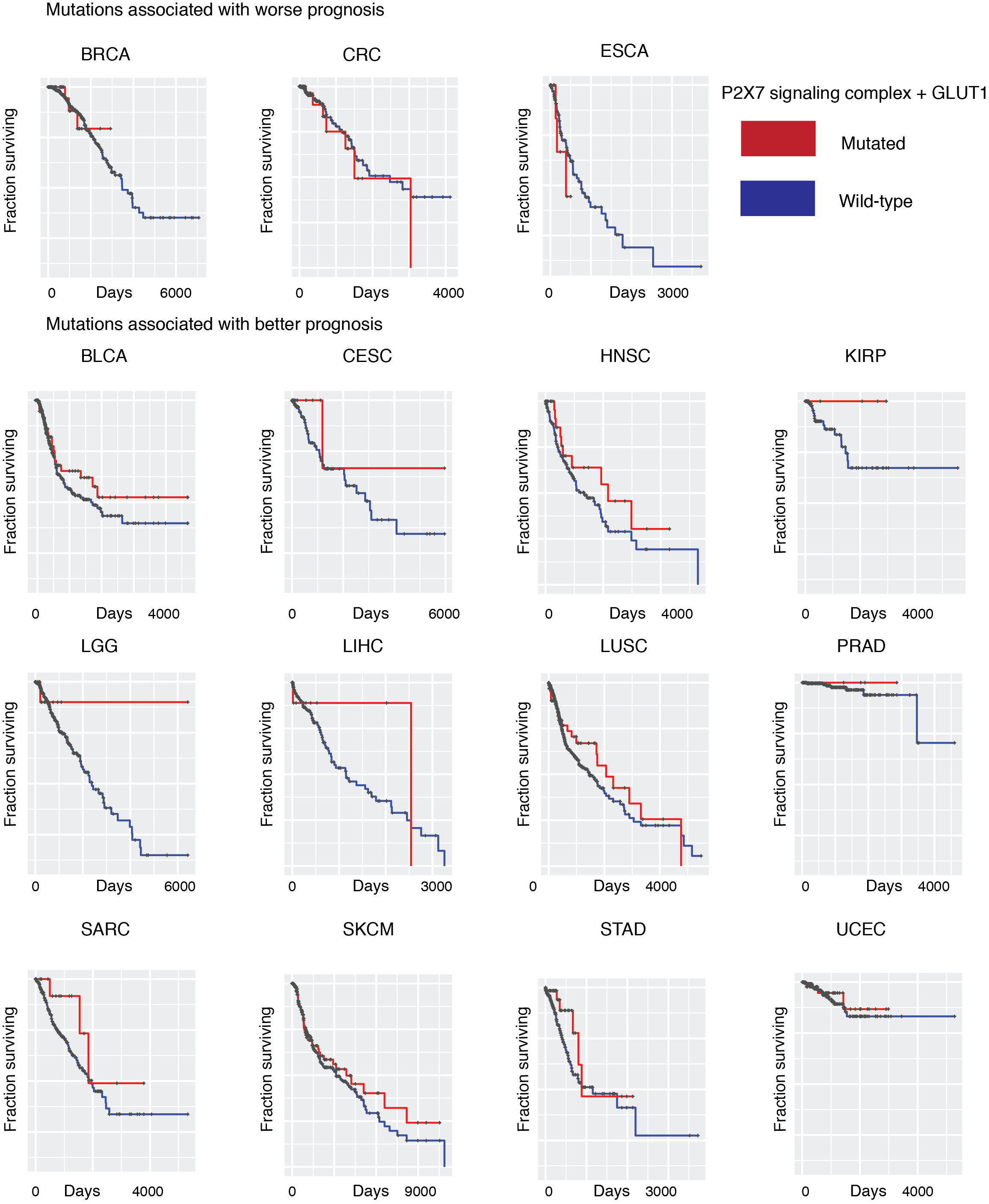


**Figure S10. Kaplan-Meier curves for 15 tumour types with mutations in the P2X7 complex.** Survival plots comparing patients having components of the P2X7 complex (or SLC2A1/GLUT1, which is regulated by P2X7) mutated versus non-mutated patients. 12 out of 15 tumour types show better prognosis when a member of the complex is mutated.


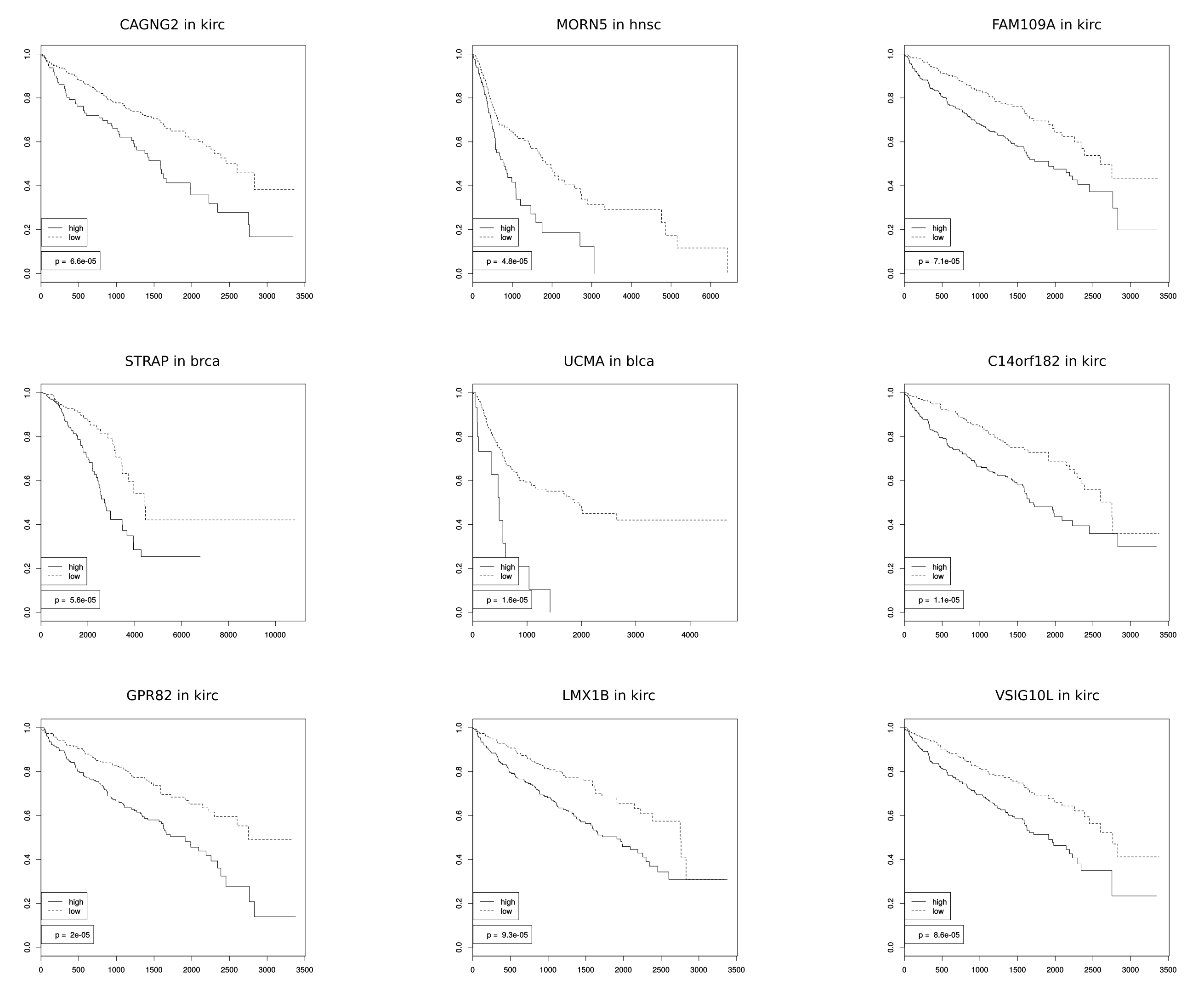


**Figure S11. Kaplan-Meier curves for 9 negatively selected genes.** Kaplan-Meier curves for nine genes found as negatively selected in cancer and showing a significant difference on survival based on the expression status.


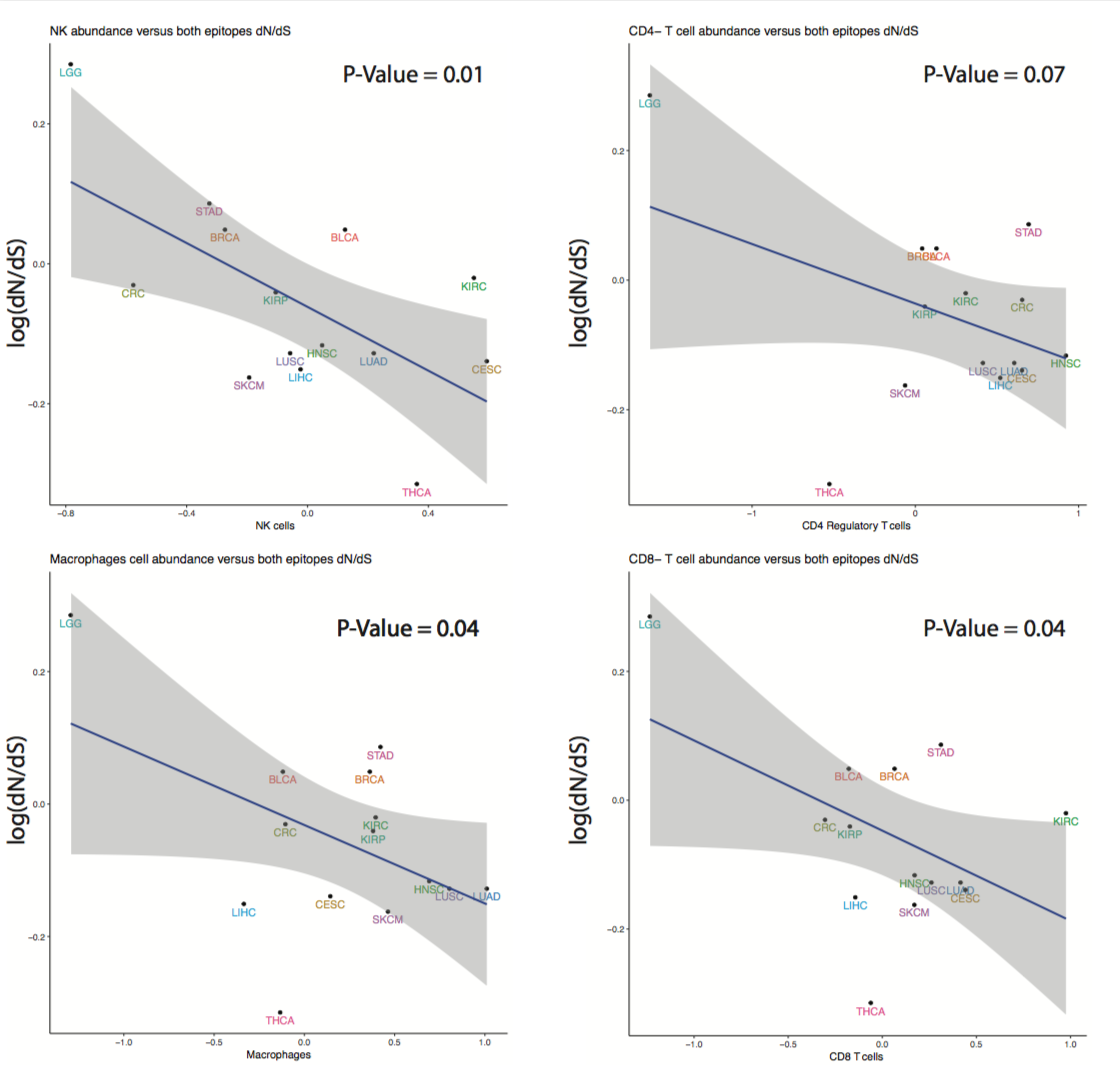


**Figure S12. Tumour type specific cytolytic activity versus dN/dS values.**

Relative abundance of different immune cell populations in different tumour types in relation to the tumour specific-dN/dS value of the MHC epitopes.


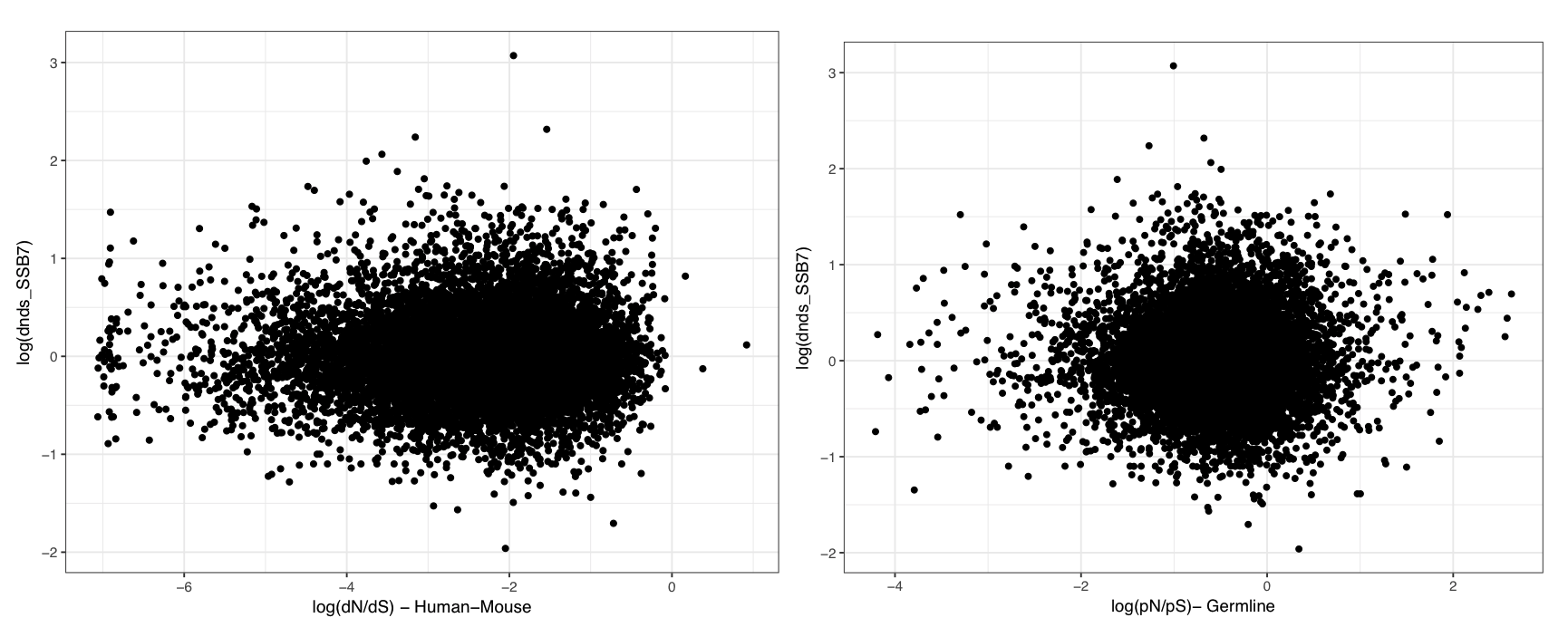


**Figure S13. dN/dS values from cancer genomes and normal human populations.**

Scatter plots of somatic dN/dS values (SSB7) and human germline dN/dS values.


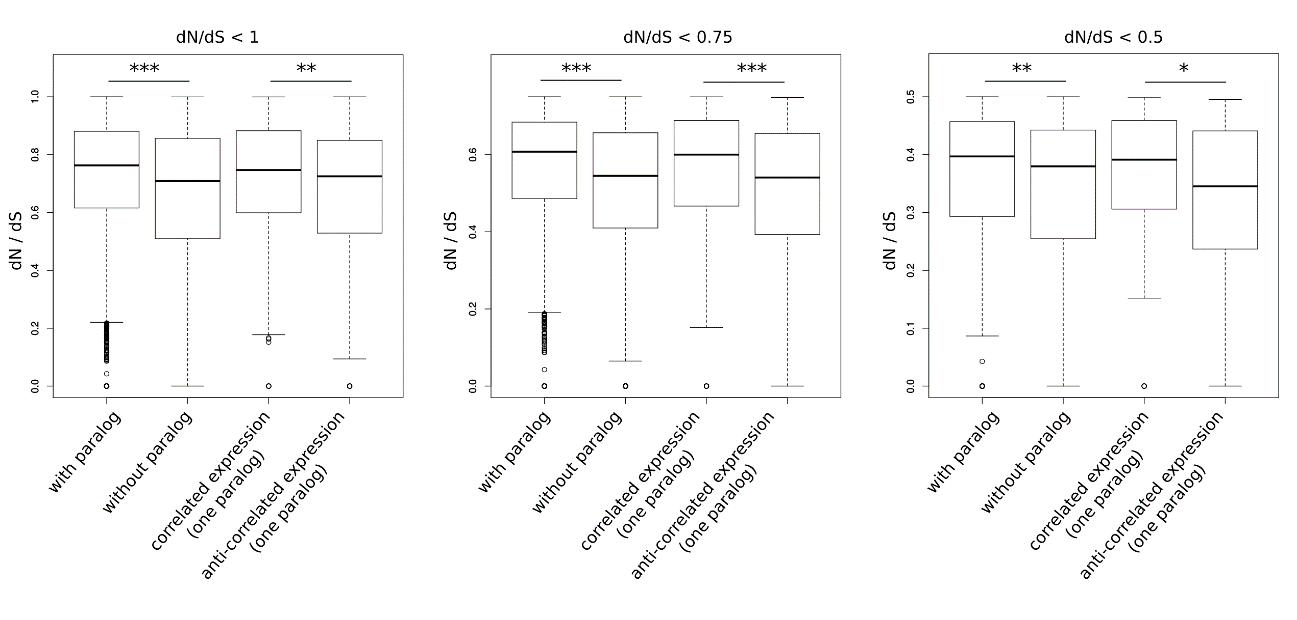


**Figure S14. dN/dS distribution of different paralog classes.**

Differences in dN/dS of genes with and without paralogs as well as of genes with one expression-correlated or one expression-anticorrelated gene are significant irrespective if genes with dN/dS < 1, dN/dS < 0.75 or dN/dS < 0.5 are considered. * = P < 0.05, ** = P < 0.01, *** = P < 0.001.

**
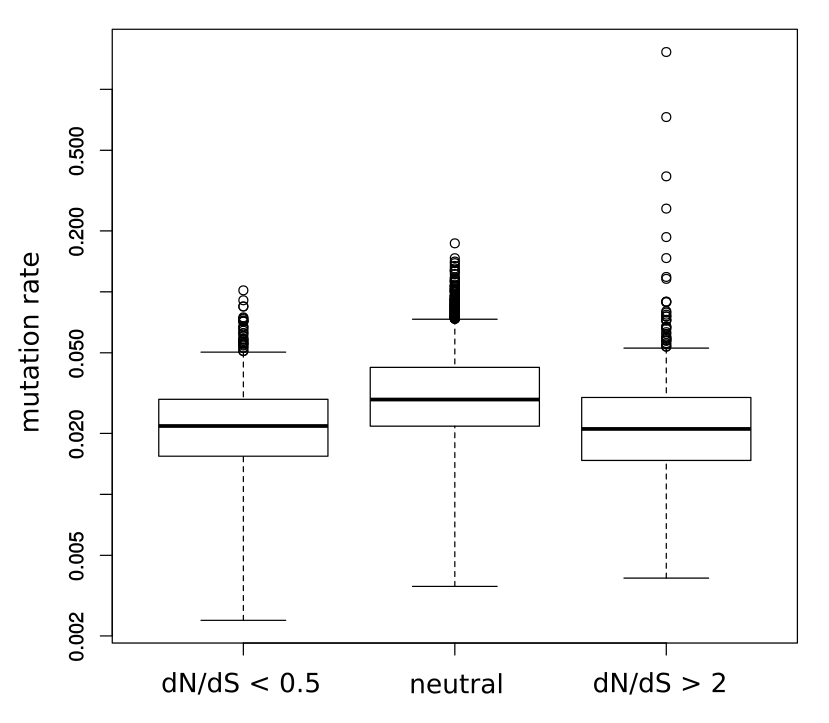
**

**Figure S15. Mutation rates of genes in different selection classes.**

The mutation rate [(synonymous + nonsynonymous mutations) / transcript length] is significantly lower for negatively selected (dN/dS < 0.5) or positively selected (dN/dS > 2) than for neutrally selected genes (0.9 < dN/dS < 1.1) (P < 0.05; Mann-Whitney *U* test). There is no significant difference between the mutation rates of negatively and positively selected genes.

**Supplemental Tables**

**Table S1. Data details for the 26 tumour types used in this study.**

Number of samples, mutations, and mutations after filtering are shown for each tumour type.

**Table S2. Results of selection test for all genes across 26 tumour types.**

Details for each gene after applying our statistical method. Top 39 genes are considered significant (FDR < 0.1).

**Table S3. Analysis results for 26 tumour types separately.**

Proportion of nonsilent and synonymous mutations separated for individual tumour types for significantly selected genes.

**Table S4. Somatic substitution bias correction results.**

dN/dS details for each tumour type before and after SSB7 correction.

**Table S5. Significantly enriched terms among negatively selected genes.** GO terms, Reactome pathways and CORUM complexes associated with negatively selected genes with a Q-value below 0.1 by GSEA are shown.

**Table S6. Tumour specific dN/dS Immune epitopes and correlation to immune activity measures.**

1. Aggregated dN/dS for epitopes combined, non-epitopes combined, and MHC-specific values. Pvalues show the proportion of times that a random value was lower than the epitope dN/dS in 1000 iterations.
2. Correlation coefficients for different measures of cytolityc activities versus dN/dS values.
3. Pvalues for paired correlations.

**Table S7. Significant genes predicted using unfiltered somatic variant lists.**

List of genes under selection (positive or negative) when removing the ABB filter (affecting the number of somatic mutations) and the expression filter (affecting the number of genes).
