# Supplementary material for: Negative selection in tumor genome evolution acts on essential cellular functions and the immunopeptidome
Source: Genome Biol. 2018 May 31;19:67. doi: 10.1186/s13059-018-1434-0 (PMC5984361; doi:10.1186/s13059-018-1434-0)
Supplement: Supplementary file 4 — This document contains the mathematical description of the method used for the context correction. It also contains the description of the simulation, the benchmarking, and the ABB score filtration. (DOCX 19 kb) [file 13059_2018_1434_MOESM4_ESM.docx]

**Supplemental Methods**

- 1. **dN/dS correction formulation**

Cancers evolve by accumulating somatic mutations which can be produced by endogenous or exogenous mechanisms. Recently, it has been demonstrated that these mechanisms are related to more than 30 mutational signatures imprinted on the cancer genome (Alexandrov et al 2013). These signatures have a particular composition of somatic substitutions which can differ from tumour to tumour. Thus, some substitutions may appear more frequently than others, such as for example UV signature in melanoma characterized by C->T changes. When estimating dN/dS values this somatic substitution bias should be taking into account, similarly as transition/transversion ratios were taking into account in models of population genetics (Yang And Bielawski, 2000). In our method we applied two models, one considering 7 and another considering 192 substitution types. To correct the *dN and dS* values for the somatic substitution bias on a gene-level, we first count the observed somatic mutations per tumour cohort and group them by substitution types. Then, the number of observed mutations per substitution type ($n_{i}$) across all genes *j* is:

$$n_{i}= \sum_{j} (n_{m,i,j}+n_{s,i,j})$$

where subscripts *m* and *s* indicate missense and synonymous substitutions respectively.

Then, the total number of mutations in the cohort is simply:

$$n= \sum_{i} n_{i}$$

And the observed rates per substitution type is:

$$r_{i}= n_{i}/n$$

Next, we calculate the total number of possible substitution sites separately for non-silent (considering missense and nonsense together) and synonymous changes and for each substitution type *i* across all genes. This value is obtained directly from the coding sequence of the genes as described in classical *dN/dS* studies (Yang And Bielawski, 2000) but counting separately each substitution type. The total number of possible substitution sites for non-silent ${(N}_{m, i, j})$ and synonymous mutations ${(N}_{s, i, j})$ per substitution type *i* across all genes *j* is:

$$N_{m,i}= \sum_{j} N_{m,i,j} and N_{s,i}= \sum_{j} N_{s,i,j}$$

And the total number of non-silent and synonymous sites across all substitution types is:

$$T_{m}= \sum_{i} N_{m,i} and T_{s}= \sum_{i} N_{s,i}$$

We can calculate an expected rate per substitution *i:*

$$p_{i}=(N_{m,i}+ N_{s,i})/(T_{m}+ T_{s})$$

The correction factor per substitution type in a given cohort is:

$$F_{i}=r_{i}/p_{i}$$

and the corrected total sites per substitution type *i* per gene is

$$N_{s,i,j}^{'}=F_{i}*N_{s,i,j}$$

$$N_{m,i,j}^{'}=F_{i}*N_{m,i,j}$$

Finally, the corrected dN/dS per gene *j* is calculated as the ratio between:

${dN}_{j}= \sum_{i} (\frac{n_{m,i,j}}{N_{m,i,j}^{'}})$ and ${dS}_{j}= \sum_{i} (\frac{n_{s,i,j}}{N_{s,i,j}^{'}})$

The dN/dS correction formula can be applied using various substitution type matrices, e.g. strand-specific single nucleotide changes (6 possible types), strand-specific single nucleotide changes with separated CpG counts (SSB7), or strand-specific trinucleotide substitutions (SSB192). In our method we have implemented the latter two models.

**1.2 Statistical test for significance**

To test against the null hypothesis of no selection (neutral evolution) for each gene, we transformed SSB-corrected *dN/dS* values to a test statistic following the model of Greenman et al 2006 (*j* is omitted as a subscript in this representation to simplify the formula):

$$U=\sum_{i} n_{m,i}-\{\sum_{i} n_{i}*\left( \frac{\sum_{i} N_{m,i}^{'}}{\sum_{i} N_{m,i}^{'}+ \sum_{i} N_{s,i}^{'}} \right)\}$$

$$V= \sum_{i} n_{i}*\{\sum_{i} N_{m,i}^{'}*\left( \frac{\sum_{i} N_{s,i}^{'}}{\left( \sum_{i} N_{m,i}^{'}+ \sum_{i} N_{s,i}^{'} \right)^{2}} \right)\}$$

$$test.score=U^{2}/V$$

Differently to a recently published method (Martincorena et al 2017), our test for selection is based on an alternative class of test statistics named score tests, based on the first derivative U of the log-likelihood at the null (For details see section test of selection in (Greenman et al 2006). The resulting approximate P-Value is computed from the chi-square distribution (df = 1) and then multiple testing-adjusted using Benjamin & Hochberg for multiple test correction. After the test, to calculate dN/dS values per gene and avoid extreme values at the gene level (0 or infinite given by dN or dS equal to 0), we added a single estimate to the synonymous and the nonsilent counts based on the composition of the gene and keeping the neutrality ratio of one between dN and dS.

**2.1 Simulation of somatic evolution using mutation frequencies**

To simulate a neutral accumulation process of somatic mutations: first, we calculated the observed frequencies of mutations by trinucleotide context in the Pancan26 cohort. As a result, each of the 192 (or 7) substitutions has an associated probability based on its occurrence in the cohort. Second, we estimated the frequency composition of trinucleotides for each of the 18034 transcripts. We then simulated somatic mutations based on the probabilities of occurrences in the cohort and the trinucleotide composition of each gene.

For each simulated mutation with its respective trinucleotide context we sampled a transcript based on its sequence composition. For example, if we want to place a TTT->TCT substitution in the exome, each transcript has an associated probability to carry the trinucleotide TTT based on its sequence composition and the total number of TTT trinucleotides in the exome. Finally, we randomly assign the mutation to one TTT within the transcript. As a consequence, there are as many TTT->TCT substitutions in the simulated as in the original dataset and we obtain a simulated cancer cohort harbouring the same mutation signatures as the original cohort. We provided the script for simulation in the synapse repository syn10464326. We simulated cohorts having 100K, 300K, 500K and 1M somatic mutations, an example file of each simulated cohorts can be obtained from synapse syn11615615.

**2.2 Annotation of simulated mutations**

The second step consisted in annotating the simulated mutations. We used the variant effect predictor v89 (variant_effect_predictor.pl -i X.muts -o X.annotated --cache --all_refseq --force_overwrite -fork 4 --assembly GRCh37 --buffer_size 15000 --pick --symbol --port 3337 --no_stats --fasta Homo_sapiens.GRCh37.dna.primary_assembly.fa) to obtain the functional annotation for each somatic substitution. We removed all variants annotated as: UTR|downstream|intron|miRNA|frameshift|non_coding|splice_acceptor_variant|splice_donor_variant|upstream|incomplete|retained. Finally, we consider mutations as silent if they are labelled as synonymous and as nonsilent otherwise excluding the noncoding as stated previously.

**2.3 Calculate dN/dS and test for selection using uncorrected, SSB7-, and SSB192-corrected.**

To estimate the number of significantly selected genes, we obtained the total number of nonsynonymous and synonymous sites for each transcript. Then, we corrected the total number of nonsynonymous and synonymous sites using the formula described in section 1.1. As mentioned before, we can use this formula to correct for any number of substitution rate parameters. Finally, we applied the statistical test described in section 1.2 to identify significantly selected genes in the simulated datasets with 100k, 500k, 1M and 3M somatic variants.

**2.4 Benchmarking using precision and recall**

To assess if our statistical framework is able to capture positively and negatively selected genes we calculated the precision and recall using a simulated dataset. First, we simulated a neutral dataset of 1M somatic mutations as described in section 2.1 and randomly selected 500 genes where we double the number of missense mutations, and 500 genes for which we divided the number of missense mutations in half. Of these 500 positively and 500 negatively selected genes we only considered genes for benchmarking for which the increase of nonsilent mutations results in a dN/dS higher than 2, and the decrease of nonsilent mutations results in a dNdS lower than 0.5, respectively. We repeated the simulation 100 times to estimate an average precision and recall for both positively and negatively selected genes. Similarly, we compared the results when using the trinucleotide context (SSB192) instead of the 7 parameters used in this study (SSB7).

To determine the number of somatic mutations needed to detect positive or negatively selected genes we simulated selected genes as previously described using 100K, 300K, 500K, 1M and 3M somatic mutation datasets. The results of the power analysis (precision and recall) at different number of somatic mutations are summarized in supplemental figure 2.

**3.1 ABB score filtration**

The Allele Balance Bias (ABB) score is a variant callability model able to identify genomic positions affected by systematic sequencing or alignment errors using human whole-exome sequencing (WES) data.  The statistical model of ABB is based on the analysis of recurrent biases in the allele balance (read fraction showing the alternative allele) at a focal position across thousands of germline WES datasets. Positions with recurrent allele balance bias are considered highly prone to systematic analysis errors and hence masked for somatic variant prediction. The fraction of somatic mutations filtered by this method is typically lower than 1% (empirical value based on the analysis of several thousand tumour-normal pairs from TCGA and ICGC). A manuscript describing ABB is in preparation.

**Reference**

Alexandrov, L.B., Nik-Zainal, S., Wedge, D.C., Aparicio, S.A., Behjati, S., Biankin, A.V., Bignell, G.R., Bolli, N., Borg, A., et al. Signatures of mutational processes in human cancer. Nature. 2013; 500:415-421.

Lawrence MS, Stojanov P, Polak P, Kryukov GV, Cibulskis K, Sivachenko A, et al. Mutational heterogeneity in cancer and the search for new cancer-associated genes. Nature. 2013;499:214-218.

Yang Z, Bielawski JP. Statistical methods for detecting molecular adaptation. Trends Ecol Evol. 2000;15:496-503.

Greenman C, Wooster R, Futreal PA, Stratton MR, Easton DF. Statistical analysis of pathogenicity of somatic mutations in cancer. Genetics. 2006;173:2187-98.

Martincorena I, Raine KM, Gerstung M, Dawson KJ, Haase K, Van Loo P, et al. Universal patterns of selection in cancer and somatic tissues. Cell. 2017;171:1029-1041.e21.

Weghorn D, Sunyaev S. Bayesian inference of negative and positive selection in human cancers. Nat Genet. 2017;49:1785-8.
