# Supplementary material for: Negative selection in tumor genome evolution acts on essential cellular functions and the immunopeptidome
Source: Genome Biol. 2018 May 31;19:67. doi: 10.1186/s13059-018-1434-0 (PMC5984361; doi:10.1186/s13059-018-1434-0)
Supplement: Supplementary file 5 — Reviewer reports and Author’s response to reviewers. (DOCX 53 kb) [file 13059_2018_1434_MOESM5_ESM.docx]

**First round of review**

**Reviewer 1**

**Are the methods appropriate to the aims of the study, are they well described, and are necessary controls included?**
see comments to authors

**Are the conclusions adequately supported by the data shown?**

see comments to authors

**Are sufficient details provided to allow replication and comparison with related analyses that may have been performed?**

see comments to authors

**Does the work represent a significant advance over previously published studies? Please explain.**

yes it's an advance. The significance of the advance really depends on the final form of the paper.

**Is the paper of broad interest to others in the field, or of outstanding interest to a broad audience of biologists?**

No

**Comments to authors**

Zapata et al. present a survey of negatively selected genes in cancer, and further characterize these genes with respect to consequence of mutations, gene function, gene redundancy, and immune-exposure. They have developed and deployed an improved approach to correct dN/dS measures, accounting for substitution biases present in different datasets. This is a useful contibution to the field, but the paper could be strengthened by attention to the issues noted below.

1.	In general, more careful attention to terminology would improve the readability of the paper. For example, when referring to detrimental mutations, it should be specified in each case if the detriment is to the tumor or the host. It is not helpful to introduce new terms that have no accepted statistical meaning, such as "recall" (page 6) in place of "power".

2.	The term "negative selection" is used very loosely. It's a paucity of mutations in genes that cannot tolerate them. This is sometimes conflated in the manuscript with the concept of driver mutations, for example on page 9, lines 11-14.

3.	Presumably genes that can tolerate mutations in cancer cells also can't tolerate them in healthy cells. How do negatively selected genes from this analysis relate to genes subjected to purifying selection in the human lineage. Likewise, for the "immune-exposed" content.

4.	How are negative and positive selection signals within the same gene accounted for? For example, a hotspot driver mutation could give a strong signal of positive selection that masks negative selection acting elsewhere in the same gene. This would be expected to contribute to the difficulty in assessing negative selection signatures in cancer.

5.	When assessing paralogs, attention should be paid not just to whether they are co-expressed, but also whether they are/are not subfunctionalized.

6.	In the immune analysis, how are epitope boundaries defined? Accounting for each patient's HLA type, as in Rooney et al., would strengthen this aspect of the study.

7.	Rational for the sudden pivot to analyzing gene expression levels and survival (page 15) should be provided.

8.	The authors directly compare to Martincorena et al. and claim superiority, mainly based on results from simulated neutral datasets having a dN/dS ratio (by either method) significantly larger than 1. However, it appears Martincorena et al. also performed testing on a simulated neutral dataset and showed no such effect. Can this discrepancy be addressed?

9.	Another comparison is done to the CBaSe method (Weghorn et al.), and results between it and SSB are mostly consistent. In fact, the method presented here only detected 5/9 genes detected by CBaSe. What improvement or benefit is there about the method presented here?

10.	Page 14 line 4-6: Is it concerning that the complex under strongest negative selection is not statistically significant after multiple testing correction? How much weight can we put on this result?

11.	Page 14 line 14-22: Were any of the HR significant on their own? Can you justify this statistical test when the confidence intervals for each HR are lost when doing the subsequent Binomial test?

12.	Page 16 line 13-17: This ignores the fact that many mutations can cause changes in peptide binding (either creating a new epitope from sequence which was not presented previously, or causing a self-peptide that was presented to no longer be presented). Is it possible that, using mutant-peptide-specific binding predictions, the results here would change?

13.	Page 17 line 22 - page 18 line 11, Fig 3d: it is puzzling to show NK cell-based features here, as NK cells are part of innate immunity and their activity is not targeted against neoantigens. The correlation shows increased cytolytic activity with lower dN/dS, or more negatively selected genes, which would make sense for cells of the adaptive immune system (CD8+ or CD4+ T cells, for example, recognizing these displayed mutations).

14.	Page 24 line 5-6: There is an official release of the TCGA MAF file (not from Dropbox, but from NCI Genomic Data Commons Data Portal), which is updated as samples get blacklisted due to changes in consent, that should be used.

15.	Page 25 line 12: Some explanation of why these 7 substitutions were chosen would be useful. Why only these 7?

16.	Page 33 line 1-7: This is set up as describing how HLA alleles present in less than 1% of Caucasian populations were used, yet goes on to state that all alleles in Shukla et al. were used, and then those with minimal set of binding peptides overlap with HLA-A0201 were selected. This does not necessarily have any relation to frequency. Inconsistent with stated objective.

17.	Figure 1A: This figure is confusing and could be reworked for clarity. The distinction between substitution sites and mutations is confusing - which is obtained from the MAF file?

18.	Figure 2B, 2C: unclear why these two genes (of the 9) chosen to be displayed. Instead, could all HRs (with confidence intervals) be shown for all 9 genes?

19.	Figure 3A: This figure is either incorrect or too difficult to understand - it shows mutations from the tumor being attached to free-floating T cells. T cells only recognize mutated peptides bound to MHC on the surface of tumor cells, the peptide does not detach and bind to the free-floating T cells. This needs to be fixed, or have the intent explained.

20.	Page 6 line 14: The explanation of "nonsynonymous sites" could be improved - the meaning was not clear for someone unfamiliar with this concept without reading the methods section. Even then (Page 25 line 12-15), is my understanding correct that the total number of nonsynonymous sites is based on all theoretically possible mutations in a transcript, and not on the observed mutations from the MAF file? What is meant by the phrase "approximate methods"?

21.	Page 7 line 3-6: States the simulated sets of negatively selected genes were made, described in Methods, but explanation of this simulation is buried within 2.4 Benchmarking and power analysis of supplemental methods. Hard to find.

22.	Page 8 line 8: It is not clear why there are two correlation estimates presented here without reading the supplemental figure legend (there are two methods in Martincorena et al.).

23.	Page 9 line 25 - page 10 line 1: Could you not just use damage scores to identify negatively selected genes?

24.	Page 17 line 13-20: Why might it be that there are different epitopes (MHC-I vs MHC-II) being negatively selected in different tumor types?

25.	Page 19 line 14-16: Could cite recent paper by Marty et al. (MHC-I Genotype Restricts the Oncogenic Mutational Landscape, Cell 2017) here.

26.	Page 32 line 8: What threshold was used for NetMHC-4.0 predictions?

27.	Supplemental methods 1.1 "Recently, it has been demonstrated that these mechanisms are related to more than 30 mutational signatures imprinted on the cancer genome" - This should be cited.

28.	Page 6 line 16, and throughout the manuscript, could some notation be used for when the dN/dS is referring to the SSB-corrected version vs when it isn't? Perhaps SSB(dN/dS) or (dN/dS)SSB? Otherwise it can be unclear which version of the measure is being used.

29.	Page 7 line 21-22: There is no introduction of the two methods (SSB7 and SSB192) by name before this point, so it is a bit confusing at this point. Perhaps a brief explanation could occur here.

30.	Page 8 line 6: Again, dNdScv is brought up without context, must infer that it is the method in Martincorena et al. Could use some explanation.

31.	Page 9 line 12: There is some ambiguity in the meaning of "opposite". Does this mean that there is accumulation of mutations that have low functional impact? Or no accumulation of mutations that have high functional impact? Subtle difference.

32.	Page 9 line 15: What is the acronym CADD?

33.	Page 11 line 18: What is a pLI score?

34.	Page 16 line 12-13: It would be good to state the source (IEDB) here, and to state the number of epitopes (13,422) in the appropriate methods section (ie. Have both pieces of information in both places).

35.	Page 20 line 10: Missing word: "demonstrating that the number of negatively selected genes [identified] depends on…" - the actual number doesn't change based on the method used, only the number identified.

36.	Page 21 line 21-23: this is an awkward sentence.

37.	Page 29 line 5-9: awkward sentence

38.	Page 29 line 11: Why not cite (16) by name (as in previous section for Martincorena et al.)? Be consistent.

39.	Page 31 line 19-20: awkward wording.

40.	Page 32 line 11: "neither" --> "not"

41.	Supplemental methods 1.1: The final corrected dN/dS per gene j formula is missing a character in the final summation for dSj.

**Reviewer 2**

**Are the methods appropriate to the aims of the study, are they well described, and are necessary controls included?**

Yes

**Are the conclusions adequately supported by the data shown?**

Yes

**Are sufficient details provided to allow replication and comparison with related analyses that may have been performed?**

Yes

**Does the work represent a significant advance over previously published studies? Please explain.**

yes, negative selection is an essential topic in current cancer genomics, and has been underestimated by other studies.

**Is the paper of broad interest to others in the field, or of outstanding interest to a broad audience of biologists?**

Yes: Selection acting on cancer genomes is a hotly debated question in the field, particularly with respect to negative selection.

**Comments to authors**

In their paper 'Negative selection in tumour genome evolution acts on

essential cellular functions and the immunopeptidome', Zapata et

al. use a large cohort of 7500 exomes to detect selection in cancer

using a modfied dn/ds ratio corrected for cancer-specific mutational

biases.

The paper is well written and sound. It addresses highly relevant

questions in current cancer genomics regarding selection acting on

somatic variation.

Major comments:

The biggest single issue I have with this pan-cancer study is that

somatic copy-number alterations (SCNAs), which have major phenotypic

effects and affect the fitness effects and selectional constraints

acting on regions of the genome, have been completely ignored. I

understand, that this is an SNV-centric study, but different cancers

have very difference propensities for chromosomal instability and

SCNAs including whole-genome duplications or large regions of LOH

directly affect selection.

Now, including these changes into the paper would be out-of-scope, but

maybe the authors could some validation to see if their results are

influenced by SCNAs. For example, one could compute dn/ds ratios using

only diploid genomic regions and compare results or redo parts of the

enrichment analyses using only diploid regions. In some way, at least

in the dicussion, this should be addressed.

Minor comments:

p4. line 18: The number of truly recurrent somatic mutations (same

position, same base) is actually very low. I assume by 'recurrent' the

authors mean that the same gene is hit? I don't think the word

'recurrent' is really necessary here, might be worth removing.

p5, lines 9-11: I likely haven't seen every single paper on that topic

in the field, so the statement that exome-wide approaches in general

have 'failed' seems a bit strong. I suggest rewording.

p5, lines 20-22: In line 19 the authors use dN/dS, next sentence they

talk about the ratio of nonsynonymous substitutions to synonymous

substitutions, which is the same. The notation should be consistent.

p8, lines 13-14: 'probably explaining...', is this referring to other

studies? If so, it is speculative and should be moved to the

discussion section if possible. If not, it should be made clearer what

this sentence refers to.

p9, lines 17-20: I would have expected genes under neg. selection to

have quite damaging mutations. This is suprising and should be

discussed.

**Authors’ response to reviewers**

***Reviewer #1: Zapata et al. present a survey of negatively selected genes in cancer, and further characterize these genes with respect to consequence of mutations, gene function, gene redundancy, and immune-exposure. They have developed and deployed an improved approach to correct dN/dS measures, accounting for substitution biases present in different datasets. This is a useful contribution to the field, but the paper could be strengthened by attention to the issues noted below.***

***R: We thank the reviewer for considering our manuscript and for noting the important contribution that our work represents to the field. We are happy to answer the concerns raised and hope our reply will clarify those. The reviewer’s comments led to several new analyses and a larger Results section, we therefore decided to split its first part into two: “A somatic substitution bias-corrected dN/dS measure reveals negative selection in cancer exomes” and “Negative selection in the context of functional impact and redundancy”. See Lines 124-125 and 202.***

***1. In general, more careful attention to terminology would improve the readability of the paper. For example, when referring to detrimental mutations, it should be specified in each case if the detriment is to the tumor or the host. It is not helpful to introduce new terms that have no accepted statistical meaning, such as "recall" (page 6) in place of "power".***

***R: We apologise for the confusion caused by unclear terminology. Similar to other studies, in our analysis we have focused on selection acting on somatic variants during tumour evolution and the impact of those variants on tumour cell proliferation [1, 2]. Briefly, we always considered mutations detrimental if they hinder tumour proliferation, so the detriment is to the tumour and not to the host. We have clarified this in lines 72, and 208-211.***

***We have also noted that the word damage or damaging mutation can be a source of confusion and thus we have renamed damage to functional impact throughout all the manuscript. In our case, we obtained functional impact scores from CADD (Combined Annotation-Dependent Depletion) [3], a tool widely used to prioritize functional, deleterious, and disease-causing variants (http://cadd.gs.washington.edu/). In our work we have demonstrated that this measure also correlates with the strength of negative selection acting in tumour evolution. Specifically, we have updated the manuscript in lines 204-213 and 215-216 to further clarify the use of functional impact in the context of cancer evolution Kircher et al 2014.***

***Recall is also known as sensitivity, and precision is also known as positive predictive value. These are well-established measures of performance for algorithms or statistical tests (given a gold standard set of known true positives is available). Precision and recall are widely used in the fields of machine learning, information retrieval, pattern recognition, and generally used to benchmark the performance of classification or prediction methods. Power is mathematically equivalent to recall, but often used in different context, i.e. power is used to describe the property of a specific test (‘is a gene negatively selected’) while recall describes the performance of a test measured on large benchmark sets (‘how many true positives can I find’). In summary, both measures would be appropriate, but we think that recall fits better here, as we used a benchmark set with a large number of true positive genes. Thus, to minimise changes in the manuscript we have kept the word recall.***

***2. The term "negative selection" is used very loosely. It's a paucity of mutations in genes that cannot tolerate them. This is sometimes conflated in the manuscript with the concept of driver mutations, for example on page 9, lines 11-14.***

***R: Our definition of negative selection in cancer is based on the idea proposed by Nowell et al where a somatic mutation conferring a selective growth advantage to the cell (compared to the surrounding cells) will be under positive selection and a mutation conferring a detriment to the cell will be under negative selection [4, 5]. Thanks to sequencing technologies we can analyse a cancer cohort where the selective advantage (or disadvantage) to the cell will be observable as a particular genotype that is present more often (or less) than expected in a random neutral model (see [6]). This definition is often mixed with the unit of selection, which could be defined at different levels, at the mutation level (i.e. driver mutation), the gene level (i.e. driver gene), the cellular level (i.e. the expanding clone), or the cohort level (cancer). In our manuscript we refer to genes under positive selection (harbouring more nonsynonymous mutations than expected) as cancer driver genes and genes under negative selection (harbouring less nonsynonymous mutations than expected) as cancer essential genes.***

***We must note that negatively selected genes will not tolerate strongly deleterious mutations, but they can tolerate neutral or even mildly deleterious mutations (see [7]). In our work, to perform this analysis we must use genes with observed somatic mutations as we were otherwise not able to compute dN/dS. To address the concern of the reviewer, we have modified the manuscript accordingly by adding a new section (Lines 208-217) in which we clarify the used terminology.***

***3. Presumably genes that can’t tolerate mutations in cancer cells also can't tolerate them in healthy cells. How do negatively selected genes from this analysis relate to genes subjected to purifying selection in the human lineage. Likewise, for the "immune-exposed" content.***

***R: We thank the reviewer for addressing this issue. At first, we were expecting that essential genes under strong negative selection in the human lineage would also be under negative selection in cancer cells, reflecting essential functions of the human cell. Nevertheless, and in agreement with the observations in a recently published paper addressing this issue [8], we did not find a strong correlation between computed dN/dS values using germline variants and cancer specific variants at the gene level (new Figure S13). The low correlation (r ~ 0.1) reaffirms that the selective pressures in somatic versus organismal evolution are different [9].***

***Additionally, the computed dN/dS values using germline variants are significantly lower than in cancer tissues [8], suggesting a relaxation of purifying selection in cancer. A likely explanation is that germline variants affect all tissues and genes harbouring a germline variant likely show impaired function in at least one tissue. Genes harbouring a somatic variant in a specific tissue, on the other hand, might not have any essential function in that tissue, so they can tolerate mutations. We have now addressed these points in the discussion in a new paragraph (Lines 517-530).***

***To address the last concern of the reviewer on this point, we compared the dN/dS value of the immune exposed regions to the dN/dS value of the non-exposed regions using germline variants (obtained from http://exac.broadinstitute.org/downloads). We did not detect any difference on the selection strength between those (dN/dS ~ 0.75 for both), suggesting that the effect reported in our manuscript is specific to somatic evolution.***

***4. How are negative and positive selection signals within the same gene accounted for? For example, a hotspot driver mutation could give a strong signal of positive selection that masks negative selection acting elsewhere in the same gene. This would be expected to contribute to the difficulty in assessing negative selection signatures in cancer.***

***R: We agree with the reviewer on the cancelling effect that different regions of the same protein under different selective pressures might have on the overall genic dN/dS. In other words, the effect of strong positively selected “hotspots” can mask the effect of negative selection. Indeed, we would expect that a hotspot mutation making the protein an essential oncogene could force purifying selection in the rest of the gene as to preserve the important function of the protein for the tumour. To explore this effect, we investigated the change on the dN/dS value of KRAS after removing somatic mutations along the gene. We observed that dN/dS strongly diminishes after masking the mutations observed in the G12 hotspot but always remained above 1, likely reflecting the presence of other positions harbouring potentially activating mutations in KRAS. We observed the same pattern for other oncogenes revealing that despite the strong effect of canonical hotspot mutations, there could be multiple activating mechanisms under selection. Nonetheless, we cannot discard that for other genes, in particular those showing a neutral signal, both selective pressures are taking place. But finding them will require a systematic analysis of even larger cohorts and specific tools testing selection simultaneously in multiple domains within a protein. We decided to not add this analysis to the manuscript but we mentioned this type of local selection in the discussion (Lines 593-598).***

***In addition, we would like to note that similar to the existence of mutation “hotspots” driving most of the positive selection signal there could theoretically also be mutation “cold-spots”. These cold-spots would represent regions of high importance to the protein and could be exploited as potential therapeutic targets. Again, much larger cohorts or deep sequencing data would be required to provide enough power to interrogate negative versus positive selection in single small regions.***

***5. When assessing paralogs, attention should be paid not just to whether they are co-expressed, but also whether they are/are not subfunctionalized.***

***R: We agree that subfunctionalization should also affect selection acting on paralog pairs. To test this, we used domain composition as a proxy for subfunctionalization. We annotated paralog pairs with InterPro domains and implemented a similarity measure of the domain composition between two proteins based on the Jaccard index (lines 716-722). In lines 244-246 and Figure 2C we demonstrate that paralogs with low domain similarity (i.e. higher subfunctionalization) are under stronger negative selection that those with high domain similarity.***

***6. In the immune analysis, how are epitope boundaries defined? Accounting for each patient's HLA type, as in Rooney et al., would strengthen this aspect of the study.***

***R: The epitope boundaries were defined by the epitope database that we used in our analysis. We downloaded the IEDB database, which contains the information of the native peptide sequence and the positions within the transcript. In case the reviewer is interested we have uploaded the set of ~70000 peptides used to synapse (syn11935058).***

***We apologise for not stating clearly that we have already performed a dN/dS computation accounting for the HLA type of the patients. Initially, we analysed patients irrespective of their HLA type and surprisingly we found a significantly lower dN/dS than on the rest of the protein in our Pancancer dataset. Then, we stratified patients into carriers and non-carriers of the HLA-A0201 allele and we compared the epitope regions of carriers of the HLA-A0201 allele to the non-epitope regions of the same patients. We performed the analysis only on the HLA-A0201 patients given the number of somatic mutations falling into those HLA-A0201-binder epitopes was the largest, and therefore we could obtain a reliable estimate of dN/dS. Nevertheless, based on the recommendation of the reviewer we have now added the comparison for the dN/dS of carriers versus non-carriers of the HLA-A0201 allele. Indeed, we find a stronger negative selection of the respective binding epitopes in the HLA-A0201 patients (line 409 – 412). Accordingly, we have added the following sentence to the manuscript: “The dN/dS value for the binding epitopes of HLA-A0201 patients was 0.87 (CI:0.78-0.97), compared to 0.94 (CI:0.86-1.03) for the binding epitopes of non-HLA-A0201 patients.”***

***Besides the large number of somatic mutations necessary to obtain reliable estimates of dN/dS, performing the dN/dS analysis combining patients and all possible alleles is highly challenging due to: i) overlapping binding peptides among different HLA alleles, ii) the strength of immune mediated negative selection varies from tumour type to tumour type and from patient to patient, and iii) each patient carries a specific combination of alleles and therefore the target epitope region is different for each, thus requiring a patient-specific SSB correction model.***

***7. Rational for the sudden pivot to analyzing gene expression levels and survival (page 15) should be provided.***

***R: We updated the introduction to the analysis exploring the link between gene expression and survival by stating more clearly the rationale (paragraph 8, section “The functional role of genes under negative selection and their impact on survival”, lines 357-359).***

***8. The authors directly compare to Martincorena et al. and claim superiority, mainly based on results from simulated neutral datasets having a dN/dS ratio (by either method) significantly larger than 1. However, it appears Martincorena et al. also performed testing on a simulated neutral dataset and showed no such effect. Can this discrepancy be addressed?***

***R: As mentioned by the reviewer, Martincorena et al also performed a simulation of neutral evolution and showed the distribution of dN/dS values in Figure 3A of their paper [6]. From this figure we cannot estimate the parameters of the distribution, and therefore we cannot assess if there is a discrepancy between dndscv performance on our simulated dataset and on their simulation as the reviewer supposes. With the revised version of the manuscript, we have provided the simulated dataset in order that our Figure S2 can be easily reproduced if needed.***

***We note that differences in the results of the two methods could be partly due to the implementation of the simulation: In our work we simulated the SNVs based on context frequency and gene composition (estimated from the TCGA cohort), whereas in the study of Martincorena they simulated the counts per gene based on a binomial model. To clarify these points, we added a paragraph to the discussion section (see lines 508 – 515).***

***It is important to note that we did not try to imply general superiority of our method as compared to the one from Martincorena et al (or Weghorn et al - see below). We believe that these methods provide complementary functions of importance. However, we demonstrate that our method is better suited to detect negatively selected genes in cancer evolution. We showed that the method of Martincorena et al yields higher estimates of the dN/dS than expected on simulated neutral data (Figure S5D) as well as consistently higher dN/dS estimates than our method on real cancer data (Figure S5C and S6). This difference is only appreciated when looking at the per-gene dN/dS estimates and not the global dN/dS estimates where both methods are highly correlated.***

***Additionally, we reproduced the same correlation of dN/dS and functional impact scores with dndscv of Martincorena et al as we did using our method, showing that genes with low dN/dS (also when computed by dndscv) are depleted in mutations with high functional impact (see new Figure 2B).***

***9. Another comparison is done to the CBaSe method (Weghorn et al.), and results between it and SSB are mostly consistent. In fact, the method presented here only detected 5/9 genes detected by CBaSe. What improvement or benefit is there about the method presented here?***

***R: As mentioned in response to point 8, we believe that the methods are complementary. The five genes identified by SSB-dNdS and CBaSe are BCL2L12, TERT, AP2S1, KRI1, and TMEM214. The four genes significant in CBaSe but not in SSB are MYH6, ACSM1, ACAN, and CEP170B, although all of them have SSB-dN/dS values lower than one. In addition to the genes proposed by CBaSE our method finds 20 additional genes under negative selection. To see whether all these genes are true positives will require extensive experimental effort, thus we cannot precisely measure the benefit of using SSB-dN/dS. Conceptually, CBaSE is based on a Bayesian framework and SSB on a non-parametric frequentist framework. The underlying assumption of CBaSE is based on the presence of an expected distribution of synonymous mutations whereas our model is not. Although we cannot rule out the shortcomings of an assumption-free model we showed that it is sufficient to explain the presence of negative selection in cancer. Remarkably, we show that genes with higher probability of being under negative selection as computed by CBaSe (even when not significant) are also depleted in mutations having a high functional impact score. We illustrated this by reproducing our main findings using the methods of Martincorena et al and Weghorn et al and added the results as part of the new main Figure 2.***

***10. Page 14 line 4-6: Is it concerning that the complex under strongest negative selection is not statistically significant after multiple testing correction? How much weight can we put on this result?***

***R: As the reviewer points out the observation alone that the P2X7 complex is under negative selection, is not very convincing as it does not remain significant after multiple testing correction. However, we believe that together with the observation that mutations within the complex lead more often than expected to a survival benefit for the patient, it illustrates that even though negative selection might not be easily detectable given the current amount of cancer genomics data, there is an agreement between estimated activity levels of certain gene groups (both using mutation and expression data) and patient survival.***

***11. Page 14 line 14-22: Were any of the HR significant on their own? Can you justify this statistical test when the confidence intervals for each HR are lost when doing the subsequent Binomial test?***

***R: None of the survival differences between mutation carriers and non-carriers is significant on its own (after multiple testing correction). We state this in the revised version of the manuscript in lines 349-351. We attribute this to the low statistical power given the small set of mutation carriers. However, by requiring an absolute cox coefficient larger than 0.1, we select for cancer types with a minimum effect size. The binomial test and the used parameters are justified by the approximate equal probability for each gene of being associated with a cox coefficient above 0.1 or below -0.1.***

***12. Page 16 line 13-17: This ignores the fact that many mutations can cause changes in peptide binding (either creating a new epitope from sequence which was not presented previously, or causing a self-peptide that was presented to no longer be presented). Is it possible that, using mutant-peptide-specific binding predictions, the results here would change?***

***R: We fully agree with the reviewer: ideally the change in binding behaviour through mutations should be taken into account. A few studies started to explore this as for example the excellent work done by Marty and colleagues where they created a mutant-peptide-specific repertoire of epitopes, tested their binding affinity, and demonstrated that patient specific MHC-I alleles determine the fate of oncogenic mutations [10]. In other words, oncogenic mutations that will bind MHC-I and therefore be presented to the immune system are less likely to be observed than the same oncogenic mutations in patients that, due to a different MHC allele will not present the neo peptide to the immune system. This work complements our findings on native epitope sequences, and also supports the presence of immune-mediated negative selection from a different angle. We therefore added the respective reference to the manuscript (lines 474-481). Testing, as the reviewer suggests, if mutations that would create neo-epitopes would be avoided, or if mutations within native epitopes would decrease binding, would require an immense amount of work. First, testing the MHC-binding affinity change induced by millions of mutations will require weeks even on a large compute cluster. Second, to generate appropriate background sets, we would need to not just simulate mutational biases on DNA level but also take into account the effect a mutation would have on the folding and function on the protein. Third, this analysis would go far beyond our dN/dS-based estimates of negative selection of genes and gene/protein regions. Unless the reviewer insists, we would therefore prefer to leave this for another study.***

***13. Page 17 line 22 - page 18 line 11, Fig 3d: it is puzzling to show NK cell-based features here, as NK cells are part of innate immunity and their activity is not targeted against neoantigens. The correlation shows increased cytolytic activity with lower dN/dS, or more negatively selected genes, which would make sense for cells of the adaptive immune system (CD8+ or CD4+ T cells, for example, recognizing these displayed mutations).***

***R: We agree with the reviewer that showing only NK cells in Figure 3D is misleading. In fact, several (5 out of 14) of the features measuring cytolytic activity showed a significant correlation with dN/dS over tissues. From a technical perspective, many of the features obtained from Rooney et al were highly correlated among each other (see Table S6). Despite these possible explanations and to avoid any confusion we have updated the manuscript and included CD8 T cells in the main Figure 4 and a supplementary figure 12 showing the correlation between NK cells, macrophages, CD4 T cells, CD8 T cells, and the epitope dN/dS.***

***Nonetheless, we still think that the relationship between NK cells and the action of selection over the immunopeptidome is interesting. A possible explanation is that in addition to the known role of NK cells on innate immunity, studies have shown that NK cells have also attributes of adaptive immunity [11] and that NK cells play an important role in immunosurveillance [12].***

***14. Page 24 line 5-6: There is an official release of the TCGA MAF file (not from Dropbox, but from NCI Genomic Data Commons Data Portal), which is updated as samples get blacklisted due to changes in consent, that should be used.***

***R: We agree with the reviewer on the importance of using up to date datasets and of respecting the right of patients to change their informed consent. The MAF file we used builds upon the official TCGA data release. However, as described in Kandoth et al, and in our methods section, a large effort has been made to harmonizing sequencing data and identifiers from different cancer types and sequencing centres [13]. Moreover, in our study various quality criteria were applied to identify and remove false positive calls. For the purpose of finding negative selection a variant call set of highest quality is crucial, as false positive variants can substantially alter the signal (much more than for positive selection) and also contamination of germline variants can create false signals of negative selection.***

***To avoid any legal issues related to changes in consent, we contacted GDC, which hosts the TCGA data, and obtained a blacklist of samples from patients who withdrew their consent. None of these samples were included in the MAF file that we used. We therefore decided to keep using the initial MAF file.***

***15. Page 25 line 12: Some explanation of why these 7 substitutions were chosen would be useful. Why only these 7?***

***R: We observed that the simplest method for the estimation of dN/dS, that does not consider differences on the rate of nucleotide substitutions or a different sequence composition (Fig. 1A), results in an inadequate statistical model (Supp. Fig. 2). Thus, we decided to implement a model that takes into account the rate of somatic substitutions observed in cancer. CpG dinucleotide-specific somatic mutation rates have been demonstrated for many tumour types [14]. Thus, our choice of seven substitution types was made to account for the six possible substitutions of individual nucleotides not considering the strand (A->C,A->T,A->G,C->A,C->T,C->G) but separately counting substitutions at CpG dinucleotides. This allows us to capture the full spectrum of substitutions plus a context-specific somatic signature common to many cancer types. We also compared our results using a model of 192 substitution types, similar to the one used in Martincorena et al 2017 [6]. As the results were highly similar, we used the simplest model in the manuscript. To address the concern raised by the reviewer, we have update the manuscript in results and methods sections (see lines 157-160, 662-667).***

***16. Page 33 line 1-7: This is set up as describing how HLA alleles present in less than 1% of Caucasian populations were used, yet goes on to state that all alleles in Shukla et al. were used, and then those with minimal set of binding peptides overlap with HLA-A0201 were selected. This does not necessarily have any relation to frequency. Inconsistent with stated objective.***

***R: We thank the reviewer for noting this inconsistency. We have updated the manuscript to clarify the methodology used in this analysis in lines 845 – 846. Specifically, we have added the sentence: “From this intersected list, we selected alleles present in less than 1% of the population.”***

***17. Figure 1A: This figure is confusing and could be reworked for clarity. The distinction between substitution sites and mutations is confusing - which is obtained from the MAF file?***

***R: We have modified Figure 1 to clarify our SSB-correction strategy and the procedure to calculate dN/dS. The mutations are obtained from the MAF file and are also used to determine, which substitution frequencies should be used to correct the number of sites. The substitution sites are taken from the human exome (Ensembl Transcripts v91).***

***18. Figure 2B, 2C: unclear why these two genes (of the 9) chosen to be displayed. Instead, could all HRs (with confidence intervals) be shown for all 9 genes?***

***R: We agree with the reviewer that there is no immediate reason for showing only two of the survival plots. However, integrating all nine into the main figure makes it look overcrowded. We therefore decided to move the full set of nine survival plots to the supplement (new Figure S11).***

***19. Figure 3A: This figure is either incorrect or too difficult to understand - it shows mutations from the tumor being attached to free-floating T cells. T cells only recognize mutated peptides bound to MHC on the surface of tumor cells, the peptide does not detach and bind to the free-floating T cells. This needs to be fixed, or have the intent explained.***

***R: We apologise for the confusion. We modified the figure 3, which is now figure 4, and the caption to explain better the underlying model.***

***20. Page 6 line 14: The explanation of "nonsynonymous sites" could be improved - the meaning was not clear for someone unfamiliar with this concept without reading the methods section. Even then (Page 25 line 12-15), is my understanding correct that the total number of nonsynonymous sites is based on all theoretically possible mutations in a transcript, and not on the observed mutations from the MAF file? What is meant by the phrase "approximate methods"?***

***R: Yes, the total number of nonsynonymous (N) (as well as synonymous, S) sites is based on all theoretically possible substitutions in a transcript (a nucleotide can be substituted by any of the other 3 nucleotides). As suggested by the reviewer we have modified Figure 1 to clarify our methodology.***

***In the evolutionary literature methods for estimating dN/dS have been classified in two categories, maximum likelihood and approximate methods. The differences between these models have been discussed previously and we have also addressed these differences partially in our discussion (Lines 504-507) [15]. We initially referred to our method as an approximate method given we do not compute a maximum likelihood estimate in our calculation of dN/dS. We obtained dN/dS values by simply counting the theoretically possible substitutions (N and S) and then divide the observed mutations (MAF file) to the value of N and S to obtain dN, dS, and ultimately dN/dS. Then, we correct the estimates of N and S using the model described in the supplementary methods section and recalculate dN/dS (resulting in SSB-dN/dS). Thus, we consider our method an improved version of the classic version of approximate methods. To avoid confusion, we have removed the word approximate from the line 112 since it is generally thought that approximate methods do not correct for different substitutions rates.***

***21. Page 7 line 3-6: States the simulated sets of negatively selected genes were made, described in Methods, but explanation of this simulation is buried within 2.4 Benchmarking and power analysis of supplemental methods. Hard to find.***

***R: We agree with the reviewer and have referenced the corresponding section more clearly in line 142-143.***

***22. Page 8 line 8: It is not clear why there are two correlation estimates presented here without reading the supplemental figure legend (there are two methods in Martincorena et al.).***

***R: We apologize for the lack of clarity. Indeed, there are two methods in Martincorena et al and for consistency we showed both. We updated the manuscript referring to both methods in lines 171.***

***23. Page 9 line 25 - page 10 line 1: Could you not just use damage scores to identify negatively selected genes?***

***R: This is an interesting question. Functional impact has been used effectively to predict cancer driver genes (i.e. genes under positive selection) [2, 16]. This strategy works well because high functional impact mutations are present more frequently in driver genes than in passenger genes (under neutral selection) (see also method oncodriveFM from Gonzalez-Perez et al 2012). We also demonstrated in this manuscript that negatively selected genes carry mutations biased towards lower functional impact mutations since these are the mutations that the gene can tolerate. Thereby, we use functional impact estimates as an independent validation but we discuss now that it could be used for prediction as well (see lines 527-530). However, as negatively selected genes host by definition a small number of non-synonymous mutations, this will work only for a subset of genes and as a complement to other methods such as SSB-dN/dS.***

***24. Page 17 line 13-20: Why might it be that there are different epitopes (MHC-I vs MHC-II) being negatively selected in different tumour types?***

***R: This is a very interesting point that supports a differential role of MHC-I and MHC-II in cancer. As the classic literature states that only MHC-I alleles are expressed ubiquitously in the body and MHC-II only in particular cell lineages, our initial expectation was to not find any association of negative selection to MHC-II binding peptides. Nonetheless, recent studies have challenged this classic view of MHC-II and provide evidence of an active role of MHC-II in tumorigenesis [17]. Understanding why MHC-I and MHC-II epitopes are being under different selective pressures in different tumour types would require a large experimental effort. As performing these experiments would be out of scope, we can only discuss some possible explanations (Lines 567-571): First, the expression levels of MHC genes varies among tissues, thus we would expect that in tumour types where we observe immune-mediated negative selection, MHC-I or MHC-II genes should be expressed (either in the tumour or the microenvironment). Second, the general levels of tissue-specific immunogenicity would determine the amount of negative selection observed, i.e. the brain is a known immune-privileged area so we don’t expect the action of negative selection, whereas skin is highly immunogenic and we would expect strong immune-mediated negative selection. Third, there is an apparent relationship between virus-mediated cancers such as liver or head and neck tumours and MHC-II specific response, suggesting that foreign peptides are being processed, and then inducing an MHCII-based immune attack. We recognise that further analysis for each case would be extremely interesting and could be addressed in a follow up study.***

***25. Page 19 line 14-16: Could cite recent paper by Marty et al. (MHC-I Genotype Restricts the Oncogenic Mutational Landscape, Cell 2017) here.***

***R: We thank the reviewer for pointing us to this paper. Indeed, the findings of this study agree with what we have presented here. We modified the sentence in lines 474-477 to acknowledge and discuss the findings of this paper and its relation to our work.***

***26. Page 32 line 8: What threshold was used for NetMHC-4.0 predictions?***

***R: We used the default parameters of netMHC-4.0, a rank threshold of 2 and 0.5 for strong and weak binders, respectively. Using both strong and weak binders resulted in a substantially larger set of variants and hence power to detect negative selection as compared to using only strong binders. We indicate these thresholds in the updated version of the manuscript (lines 824-825).***

***27. Supplemental methods 1.1 "Recently, it has been demonstrated that these mechanisms are related to more than 30 mutational signatures imprinted on the cancer genome" - This should be cited.***

***R: We have added the Alexandrov et al 2013 citation to the paragraph.***

***28. Page 6 line 16, and throughout the manuscript, could some notation be used for when the dN/dS is referring to the SSB-corrected version vs when it isn't? Perhaps SSB(dN/dS) or (dN/dS)SSB? Otherwise it can be unclear which version of the measure is being used.***

***R: We apologise for the ambiguous use of abbreviations and thank the reviewer for the suggestion. We have modified the manuscript in the first section of the results lines 128. Also, we have specified if we are referring to SSB-dN/dS or to other dN/dS method explicitly in the rest of the manuscript.***

***29. Page 7 line 21-22: There is no introduction of the two methods (SSB7 and SSB192) by name before this point, so it is a bit confusing at this point. Perhaps a brief explanation could occur here.***

***R: We have modified the manuscript in lines 157 to 160 to introduce the methods prior to the first usage of the abbreviation.***

***30. Page 8 line 6: Again, dNdScv is brought up without context, must infer that it is the method in Martincorena et al. Could use some explanation.***

***R: We clarified in the manuscript that dNdScv and dNdSloc are the methods of Martincorena et al. Please see answer to point 22 and Lines 170-171.***

***31. Page 9 line 12: There is some ambiguity in the meaning of "opposite". Does this mean that there is accumulation of mutations that have low functional impact? Or no accumulation of mutations that have high functional impact? Subtle difference.***

***R: We have rewritten this section in lines 204 – 211 to clarify that we expect a depletion of high functional impact mutations in genes under negative selection. For genes not influenced by selection we expect no bias.***

***32. Page 9 line 15: What is the acronym CADD?***

***R: CADD scores (Combined Annotation-Dependent Depletion) estimate the functional impact or damage of a mutation on a protein [3]. We now spell out the abbreviation at its first instance and better explain the rationale behind using it (see also our answer to point 1).***

***33. Page 11 line 18: What is a pLI score?***

***R: Thanks for pointing us at this oversight. We have now included the definition of pLI in the manuscript. In brief, pLI score is the probability of a gene to be Loss-of-function (LoF) Intolerant [18]. It measures the probability of a strong phenotypic impact if the loss of function mutation occurs in one copy of a gene. That means genes having a low pLi score can harbour mutations in one copy without affecting the phenotype of the cell due to redundancy (but could still show a strong recessive phenotype if both alleles are mutated). Higher pLI scores indicate that LoF mutations in one allele of a gene will have a dominant effect on the phenotype. We have modified lines 266-268.***

***34. Page 16 line 12-13: It would be good to state the source (IEDB) here, and to state the number of epitopes (13,422) in the appropriate methods section (ie. Have both pieces of information in both places).***

***R: We modified the manuscript as suggested in lines 395-396.***

***35. Page 20 line 10: Missing word: "demonstrating that the number of negatively selected genes [identified] depends on…" - the actual number doesn't change based on the method used, only the number identified.***

***R: We added the word “identified” as suggested by the reviewer (Line 500).***

***36. Page 21 line 21-23: this is an awkward sentence.***

***R: We removed the sentence (line 551).***

***37. Page 29 line 5-9: awkward sentence***

***R: We have updated the sentence (lines 751-754)***

***38. Page 29 line 11: Why not cite (16) by name (as in previous section for Martincorena et al.)? Be consistent.***

***R: We agree and have updated the manuscript in line 756.***

***39. Page 31 line 19-20: awkward wording.***

***R: We modified the sentence in order to better explain the performed test (lines 810-811).***

***40. Page 32 line 11: "neither" --> "not"***

***R: We replaced the word “neither” by “not” as suggested by the reviewer (lines 828).***

***41. Supplemental methods 1.1: The final corrected dN/dS per gene j formula is missing a character in the final summation for dSj.***

***R: We have added the missing character to the supplementary methods.***

***Reviewer #2: In their paper 'Negative selection in tumour genome evolution acts on essential cellular functions and the immunopeptidome', Zapata etal. use a large cohort of 7500 exomes to detect selection in cancer using a modified dn/ds ratio corrected for cancer-specific mutational***

***Biases. The paper is well written and sound. It addresses highly relevant questions in current cancer genomics regarding selection acting on somatic variation.***

***Major comments:***

***The biggest single issue I have with this pan-cancer study is that somatic copy-number alterations (SCNAs), which have major phenotypic effects and affect the fitness effects and selectional constraints acting on regions of the genome, have been completely ignored. I understand, that this is an SNV-centric study, but different cancers have very difference propensities for chromosomal instability and SCNAs including whole-genome duplications or large regions of LOH directly affect selection.***

***Now, including these changes into the paper would be out-of-scope, but maybe the authors could do some validation to see if their results are influenced by SCNAs. For example, one could compute dn/ds ratios using only diploid genomic regions and compare results or redo parts of the enrichment analyses using only diploid regions. In some way, at least in the discussion, this should be addressed.***

***R: We thank the reviewer for this suggestion and fully agree about the importance of copy number alterations on the selection constraints present in the cancer cell. The effect of gene duplication on the fitness of an individual or a population has been largely debated [19]. The classic view portrayed by Susumu Ohno implies that the creation of redundancy will allow for innovation and therefore relaxed purifying selection on the extra copies [20]. Indeed, we could demonstrate here that negative selection in tumours is relaxed in genes with paralogs (Lines 228-238).***

***To address the concern of the reviewer and to correct for potential biases given by differences on selective constrains in non-diploid regions we performed a comparison of dN/dS values in diploid-only versus all regions. We have added a section to the results and the methods in lines 257-265 and 875-882. We added a supplementary figure showing the dN/dS for genes using all mutations compared to mutations in diploid regions (Figure S9). The correlation (r) when considering all, significantly positive, and significantly negative genes was 0.86 (P-Val <10e-16), 0.95 (P-Val <10e-6), and 0.83 (P-Val < 10e-6), respectively. All but 2 genes remained significant for being under negative selection when using only SNVs in diploid regions. We have also included a new paragraph into the discussion addressing these results (Lines 517 - 530).***

***Minor comments:***

***p4. line 18: The number of truly recurrent somatic mutations (same position, same base) is actually very low. I assume by 'recurrent' the authors mean that the same gene is hit? I don't think the word 'recurrent' is really necessary here, might be worth removing.***

***R: We agree with the reviewer and removed the word “recurrent” at the indicated position (line 86)***

***p5, lines 9-11: I likely haven't seen every single paper on that topic in the field, so the statement that exome-wide approaches in general have 'failed' seems a bit strong. I suggest rewording.***

***R: Following the suggestion of the reviewer, we reformulated the sentence by saying that previous approaches “only identified a small number of negatively selected genes” and referenced the respective papers in line 103.***

***p5, lines 20-22: In line 19 the authors use dN/dS, next sentence they talk about the ratio of nonsynonymous substitutions to synonymous substitutions, which is the same. The notation should be consistent.***

***R: We thank the reviewer for noting this. Now, we first introduced the concept of dN/dS as the ratio of nonsynonymous substitutions to synonymous substitutions per site in line 112, and then refer to dN/dS (or SSB-dN/dS when we refer to our particular method – see comments to reviewer 1) throughout the rest of the manuscript. We removed the reference to dN/dS from the indicated line.***

***p8, lines 13-14: 'probably explaining...', is this referring to other studies? If so, it is speculative and should be moved to the discussion section if possible. If not, it should be made clearer what***

***this sentence refers to.***

***R: We modified the sentence by removing the speculative wording (lines 178-181).***

***p9, lines 17-20: I would have expected genes under neg. selection to have quite damaging mutations. This is surprising and should be discussed.***

***R: After the revision we have added a separate section trying to clarify the reasoning behind the absence of strongly damaging / high functional impact mutations in negatively selected genes (also see answer to reviewer 1, point 1). In short, any high functional impact somatic mutation appearing in a gene under strong negative selection will be eliminated from the genetic pool. Thus, genes under negative selection will not tolerate high impact damaging mutations and those mutations will not be observable in the dataset. As a consequence, the only mutations that we observe in negatively selected genes are those with low or none (synonymous) functional impact. For additional literature on this topic we refer the reviewer to [21, 22]. In brief, McFarland et al has analysed the relationship between the strength of deleteriousness and the presence of those mutations in cancer demonstrating that strongly deleterious mutations should be found at low frequency in cancer. In our work we have demonstrated such dependence irrespective of the method used to calculate dN/dS.***

***References:***

***1. Greaves M, Maley CC. Clonal evolution in cancer. Nature. 2012;481:306-313***

***2. Zapata L, Susak H, Drechsel O, Friedländer MR, Estivill X, Ossowski S. Signatures of positive selection reveal a universal role of chromatin modifiers as cancer driver genes. Sci Rep. 2017;7:13124.***

***3. Kircher M, Witten DM, Jain P, O'Roak BJ, Cooper GM, Shendure J. A general framework for estimating the relative pathogenicity of human genetic variants. Nat Genet. 2014;46:310-315.***

***4. Stratton MR, Campbell PJ, Futreal PA. The cancer genome. Nature. 2009;458:719-724.***

***5. Nowell PC. The clonal evolution of tumor cell populations. Science. 1976;194:23-28.***

***6. Martincorena I, Raine KM, Gerstung M, Dawson KJ, Haase K, Van Loo P, et al. Universal Patterns of Selection in Cancer and Somatic Tissues. Cell. 2017;171:1029-1041.e21.***

***7. McFarland CD, Korolev KS, Kryukov GV, Sunyaev SR, Mirny LA. Impact of deleterious passenger mutations on cancer progression. Proc Natl Acad Sci U S A. 2013;110:2910-2915.***

***8. Zhou Z, Zou Y, Liu G, Zhou J, Wu J, Zhao S, et al. Mutation-profile-based methods for understanding selection forces in cancer somatic mutations: a comparative analysis. Oncotarget. 2017;.***

***9. Sidow A, Spies N. Concepts in solid tumor evolution. Trends Genet. 2015;31:208-214.***

***10. Marty R, Kaabinejadian S, Rossell D, Slifker MJ, van de Haar J, Engin HB, et al. MHC-I Genotype Restricts the Oncogenic Mutational Landscape. Cell. 2017;.***

***11. Vivier E, Raulet DH, Moretta A, Caligiuri MA, Zitvogel L, Lanier LL, et al. Innate or adaptive immunity? The example of natural killer cells. Science. 2011;331:44-49.***

***12. Morvan MG, Lanier LL. NK cells and cancer: you can teach innate cells new tricks. Nature Reviews Cancer. 2016;16:7.***

***13. Kandoth C, McLellan MD, Vandin F, Ye K, Niu B, Lu C, et al. Mutational landscape and significance across 12 major cancer types. Nature. 2013;502:333-339.***

***14. Greenman C, Wooster R, Futreal PA, Stratton MR, Easton DF. Statistical analysis of pathogenicity of somatic mutations in cancer. Genetics. 2006;173:2187-2198.***

***15. Yang Z, Bielawski JP. Statistical methods for detecting molecular adaptation. Trends Ecol Evol. 2000;15:496-503.***

***16. Gonzalez-Perez A, Lopez-Bigas N. Functional impact bias reveals cancer drivers. Nucleic Acids Res. 2012;40:e169.***

***17. Kreiter S, Vormehr M, van de Roemer N, Diken M, Löwer M, Diekmann J, et al. Mutant MHC class II epitopes drive therapeutic immune responses to cancer. Nature. 2015;520:692-696.***

***18. Lek M, Karczewski KJ, Minikel EV, Samocha KE, Banks E, Fennell T, et al. Analysis of protein-coding genetic variation in 60,706 humans. Nature. 2016;536:285-291.***

***19. Taylor JS, Raes J. Duplication and divergence: the evolution of new genes and old ideas. Annu Rev Genet. 2004;38:615-643.***

***20. Ohno S. Evolution by gene duplication. 1970. .***

***21. McFarland CD, Korolev KS, Kryukov GV, Sunyaev SR, Mirny LA. Impact of deleterious passenger mutations on cancer progression. Proc Natl Acad Sci U S A. 2013;110:2910-2915.***

***22. McFarland CD, Mirny LA, Korolev KS. Tug-of-war between driver and passenger mutations in cancer and other adaptive processes. Proc Natl Acad Sci U S A. 2014;111:15138-15143.***

**Second round of review**

**Reviewer 1**

The authors have considered in detail what was quite a long list of initial comments and criticisms. The revised manuscript is substantialy improved and is now suitable for publication.

**Reviewer 2**

I thank the authors for carefully addressing my concerns and comments. I believe the article is an interesting contribution to understanding selection in cancer.
